# SNHG family lncRNAs promote Ewing sarcoma cell fitness through intronic snoRNA-guided rRNA modifications

**DOI:** 10.1093/narcan/zcag019

**Published:** 2026-08-26

**Authors:** Marcela Briones-Martin-del-Campo, Alex G Lee, Truc Dinh, Virginie Marchand, Yuri Motorin, Marta Román, Qiuli Bi, Stanley G Leung, Max A Horlbeck, Leanne C Sayles, Bo Kyung Alex Seong, Luke Gilbert, Kimberly Stegmaier, E Alejandro Sweet-Cordero

**Affiliations:** Department of Pediatrics, University of California, San Francisco, CA 94158, United States; Department of Pediatrics, University of California, San Francisco, CA 94158, United States; Department of Pediatrics, University of California, San Francisco, CA 94158, United States; Epitranscriptomics and RNA Sequencing Core Facility, Université de Lorraine, CNRS, INSERM, IBSLor (UAR2008/US40), Nancy F-54000, France; IMoPA (UMR7365), CNRS, Université de Lorraine, Nancy F-54000, France; Department of Pediatrics, University of California, San Francisco, CA 94158, United States; Department of Pediatrics, University of California, San Francisco, CA 94158, United States; Department of Pediatrics, University of California, San Francisco, CA 94158, United States; Department of Pediatrics, Division of Genetics and Genomics, Boston Children’s Hospital, Boston, MA 02115, United States; Department of Pediatrics, University of California, San Francisco, CA 94158, United States; Department of Pediatric Oncology, Dana-Farber Cancer Institute and Boston Children’s Hospital, Boston, MA, United States; Department of Urology, University of California, San Francisco, CA 94158, United States; Department of Pediatric Oncology, Dana-Farber Cancer Institute and Boston Children’s Hospital, Boston, MA, United States; Department of Pediatrics, University of California, San Francisco, CA 94158, United States

## Abstract

Over 90% of Ewing sarcomas (EWS) are driven by the EWS–FLI1 fusion oncoprotein. EWS–FLI1 acts as a pioneer transcription factor, altering the expression of hundreds of genes including many long non-coding RNAs (lncRNAs). The role of these lncRNAs in the oncogenesis of EWS is mostly unknown. We developed a CRISPR interference (CRISPRi) strategy to knock down expression of lncRNA loci expressed in EWS and evaluate their effect on proliferation *in vitro* and tumor growth *in vivo*. While most lncRNA knockdown had minimal effect on cell proliferation, selective inhibition of SNHG1, SNHG12, and SNHG30 significantly reduced both proliferation of Ewing cells *in vitro* and the growth of Ewing tumors *in vivo*. These results were validated by individual knockdown followed by RNA-seq to assess their functional impact. We observed a decrease in post-transcriptional modifications (2′-O-methylation and pseudouridylation) at specific rRNA positions known to be guided by snoRNAs encoded in SNHG1 and SNHG12. Collectively, we demonstrate that expression of SNHG1, SNHG12, and SNHG30 in EWS cells contributes to the maintenance of cancer cell fitness via their role in intron-processed snoRNA genes. Thus, post-transcriptional regulation of rRNA may be a previously underappreciated consequence of EWS–FLI1 expression.

## Graphical Abstract

**Graphical Abstract:**
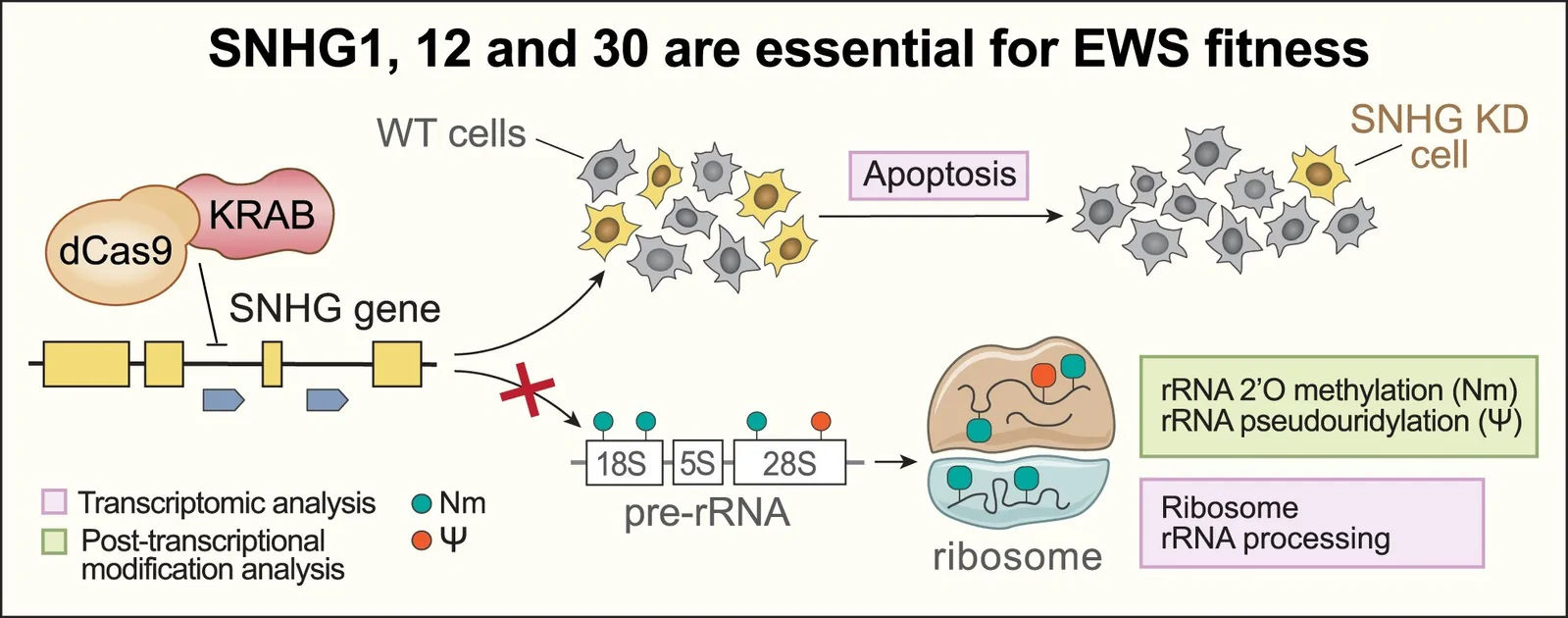

## Introduction

Non-coding RNAs (ncRNAs) are transcripts that lack protein-coding potential. ncRNAs include ribosomal RNAs (rRNA), transfer RNAs (tRNA), small nuclear RNA (snRNA), small nucleolar RNA (snoRNA), and others. Many of these ncRNAs are widely expressed and participate in a range of biological processes including protein translation and RNA modification. Other ncRNAs regulate gene expression at the epigenetic, transcriptional, and post-transcriptional level. A large class of these regulatory ncRNAs are over 200 nucleotides in length and are called long non-coding RNAs (lncRNAs). LncRNA expression is cell type-specific, and their function is linked to (i) their secondary RNA structure, (ii) subcellular localization, (iii) association with DNA enhancers, and (iv) association with chromatin-modifying proteins [1, 2]. LncRNAs have in some cases been found to contribute to the molecular pathogenesis of cancer [3, 4]. Some cancer-associated lncRNAs function as oncogenes or tumor suppressors [5] through *cis-* and *trans*-regulatory functions [6]. Understanding lncRNA function could lead to new cancer therapies [7, 8]. LncRNAs have been suggested to play a role in pediatric cancers, yet a systematic analysis of their functions is in most cases lacking (reviewed in Aryee *et al.* [9]).

Ewing sarcoma (EWS) is representative of a class of solid tumors seen mostly in pediatric and young adult patients that are driven by oncogenic fusions [10]. EWS–FLI1 is a fusion oncoprotein that functions as a pioneer transcription factor to reprogram the epigenome by recruiting *de novo* enhancers and altering gene expression [11–13]. Previously, we identified lncRNA EWSAT1 as a downstream target of EWS–FLI1 that facilitates the development of EWS via the repression of a set of target genes [14]. Other lncRNAs have also been implicated in the biology of pediatric sarcomas including EWS [9]. For example, lncRNA MALAT1 is upregulated by c-MYC and is thought to be involved in Ewing cell proliferation [15]. However, a systematic evaluation of lncRNA function in Ewing has to our knowledge not been described.

CRISPR interference (CRISPRi) can be used to repress expression of lncRNAs and thus systematically characterize their function through loss-of-function studies [16]. CRISPRi has enabled the systematic analysis of the functional role of lncRNAs in cancer [17]. Here, we used CRISPRi to evaluate the role of lncRNAs in EWS. We evaluated both lncRNAs directly regulated by EWS–FLI1 as well as lncRNAs highly expressed in this tumor type yet not directly regulated by the oncogene. Of a total of 1162 loci tested, we identified 51 loci that modify EWS cell proliferation including three genes from the snoRNA host genes (SNHG) family: SNHG1, SNHG12, and SNHG30. Repression of SNHG1, SNHG12, or SNHG30 by CRISPRi decreased proliferation of EWS cells *in vitro* and tumor growth *in vivo*. Several studies have proposed a role for SNHG1 and SNHG12 in cancer (reviewed in Zimta *et al.* [18]). The key feature of SNHG genes is that they host and generate snoRNAs from their introns through splicing [19–22]. snoRNAs are small RNAs that are involved in either 2′-O-ribose methylation or pseudouridylation of rRNAs, spliceosomal snRNAs, tRNAs, and mRNAs [23–25]. Most studies have focused on the host lncRNA genes, although recent work has highlighted the importance of the intronic snoRNAs in cancer [26]. These results demonstrate that the decrease in cellular fitness seen in SNHG1, SNHG12, and SNHG30 knockdown in EWS is due to the intronic snoRNA genes and their role in rRNA post-transcriptional modifications. Together, our findings suggest a role for snoRNA functions in the oncogenesis of EWS.

## Materials and methods

### Mice

All procedures involving mice were approved by the Institutional Animal Care and Use Committee (IACUC) at the University of California, San Francisco (protocol # AN157619). Mice were housed in the HDFCCC animal facility at UCSF. The NOD scid IL2Rg^null^ (NSG) mice used for tumor studies were obtained from Jackson Laboratory, Bar Harbor, Maine, USA (JAX Strain no. 005557). Both male and female mice were used equally. Mice were euthanized before their tumors grew to the maximum allowable tumor size of 1 cm^3^ per flank.

### Cell lines

Cell lines RDES, SKNMC, TC-32, A673, SKNEP1, EW8, SKPNDW, TTC466, SJSA, and HEK293 were obtained from ATCC. All cell lines were maintained in Dulbecco’s modified Eagle’s medium (DMEM) supplemented with 10% bovine growth serum (Hyclone, Cat. No. SH30541.03) and 1% penicillin-streptomycin-glutamine. TC-71 was a gift from Timothy Triche (University of Southern California, Los Angeles, California, USA). PID285, PID290, and PID799 are Patient Derived Xenograft Cell (PDXC) lines that were generated from PDXs established from Ewing patients and were cultured in IMDM containing 10% fetal bovine serum (FBS) and 1% penicillin-streptomycin-glutamine. EWS502 cells expressing the degradable N-dTAG EWS–FLI1 (FKBP12^F36V^-fused at N-terminal of EWS–FLI1) were generated as previously described [27]. The hetero-bifunctional small molecule dTAG^VHL^ was synthesized as previously described [27]. A673 shEWS–FLI1 and shGFP cell lines were generated as previously described [14]. All cells were incubated at 37°C in 5% CO_2_. Cells were routinely tested to exclude *Mycoplasma* contamination. Comparison of STR testing (IDEXX BioAnalytics) on genomic DNA from our cell lines to ATCC data confirmed the identity of the cell lines.

### Plasmids and cloning

For CRISPRi-dCas9-KRAB knockdowns, sgRNAs targeting different loci (Integrated DNA Technologies) were cloned into the lentiviral vectors pU6-sgRNA EF1alpha Puro-T2A-GFP (pLG20) and the mCherry-containing version pLG21 (gifts from Dr Luke Gilbert, UCSF). See Supplementary Table S1 for sgRNA sequences. We used two different plasmids for dCas9-KRAB expression: UCOE-EF1a dCas9-BFP-KRAB (a gift from Dr Luke Gilbert, UCSF) and doxycycline-inducible (dox-inducible) pHAGE TRE dCas9-KRAB (Addgene, Cat. No. 50917). For SNHG1 overexpression, two distinct DNA constructs of SNHG1 were cloned into pSBbi-RP (Addgene, Cat. No. 60515) using the In-Fusion Snap Assembly Master Mix (Takara, Cat. No. 638947). The construct named “cDNA SNHG1” contains the complementary DNA SNHG1 (NCBI RefSeq transcript variant 1, NR_003098.2) amplified by polymerase chain reaction (PCR) from plasmid pLenti-GIII-CMV-RFP-2A-Puro-cDNA_SNHG1 (Applied Biological Materials Inc, Cat. No. 44549061). The construct named “gDNA SNHG1” contains the complete SNHG1 genomic sequence amplified by PCR from genomic DNA of A673 cells. The plasmid pTS395_PhCMV-SB100 (Addgene, Cat. No. 169633), which encodes SB100 transposase was used to introduce the pSBbi-RP plasmids containing the SNHG1 DNA constructs into the A673 cells via the Sleeping Beauty transposon system [28]. Plasmids pLKO.1-shEWS–FLI1 and pLKO.1-shGFP were generated as previously described [14].

### Lentiviral infections

We generated a set of stable Ewing cell lines expressing constitutive or dox-inducible dCas9-KRAB. The parental cell lines A673, TC-71, EW8, TC-32, SJSA, and HEK293T were transduced with lentiviral vector UCOE-EF1a dCas9-BFP-KRAB, and the cell line A673 was also transduced with lentiviral vector pHAGE TRE dCas9-KRAB. Cells were BFP^+^ sorted or selected with antibiotic 2.0 mg/ml G418 Sulfate (Gibco, Cat. No. 10131035). The dCas9-KRAB-expressing cancer cell lines were infected with the following lentivirus sgRNA vectors: sgNT, sgSNHG1-1, sgSNHG1-2, sgSNHG12-1, sgSNHG12-2, sgSNHG30-1, sgSNHG30-2, and sgAURKB. We evaluated knockdown efficiency of dCas9-KRAB^+^ cell lines using a CD59-specific sgRNA. After transduction, cells were selected with 2 µg/ml puromycin, counted, and seeded for *in vitro* assays. For dox-inducible SNHG knockdowns, cells were treated with 1 μg/ml doxycycline for 6 days.

### Reverse transcription-quantitative polymerase chain reaction (RT-qPCR)

RNA was isolated using the QIAshredder homogenization (Qiagen, Cat. No. 79654) and RNAeasy column kit (Qiagen, Cat. No. 74106). Complementary DNA (cDNA) was synthesized using the Maxima First Strand cDNA Synthesis Kit (Thermo Fisher, Cat. No. K1642). RT-qPCR was performed using PerfeCTa SYBR Green SuperMix (Quanta Biosciences, Cat. No. 95054). All RT-qPCR primers are listed in Supplementary Table S2. All Ct values were normalized to housekeeping gene HPRT1.

### CRISPRi library design and construction

The CRISPRi library targets 1162 loci derived from multiple sources: (i) lncRNAs expressed in EWS tumors from RNA-seq data of 15 EWS patients from our laboratory cohort, (ii) lncRNAs controlled by EWS–FLI1 from RNA-seq data comparing A673 cell line transduced with shGFP versus shFLI1 (FC > 2.0 and FDR < 0.1) [14], and (iii) lncRNAs controlled by EWS–FLI1 from an RNA-seq experiment in EWS502 cell line that was transduced with lentivirus encoding degradable EWS–FLI1, treated with small molecule dTAG^VHL^ and cells harvested post-treatment at 6, 24, and 72 h (adjusted *P*-value <.05) (unpublished data from laboratory of Dr Kimberly Stegmaier). Although the CRISPRi library was designed to focus primarily on targeting lncRNAs, it also targets a smaller fraction of other gene types, including small RNAs, pseudogenes, and a set of protein coding genes (pcg) specifically and highly expressed in EWS tumor samples versus other diseases (from our laboratory cohort). We also included genes as positive controls: (i) lncRNA hits shared in more than three cell lines from CRINCL library [17], (ii) essential pcg found as hits in the whole-genome CRISPRi library [16], and (iii) pcg involved in Ewing’s growth from review of the literature.

We defined canonical transcription start site (TSS) and alternative TSS per loci using the FANTOM5 cap analysis of gene expression (CAGE)-based TSS annotations [29, 30] specifically in Ewing cells, SKNMC, and Hs863.T cell lines. To reduce redundancy, if a TSS was shared between Ewing data or canonical data, the CAGE peaks were merged into a single peak. We targeted one TSS for 55.5% of the genes in the library, two TSS for 25.1% of the genes, and 19.3% with three or more TSS (Supplementary Fig. S2C).

The design of sgRNAs was done using the Python-based CRISPRiaDesign pipeline (https://github.com/mhorlbeck/CRISPRiaDesign) [31]. We selected the top five candidate sgRNAs for each TSS based on the predicted activity and off-target scores. Eight-hundred five non-targeting sgRNAs matched to the base composition of the library were included as controls. Cloning (BstXI and BlpI) and PCR adapter sites flanked the 20 bp protospacer of all designed sgRNAs. sgRNAs were synthesized as a single oligonucleotide pool (Twist Bioscience), PCR amplified, and cloned into the lentiviral vector pLG21 following the cloning protocol as previously described (https://weissman.wi.mit.edu/crispr/). The final library with 9992 sgRNAs targeting 1162 loci confers puromycin resistance and mCherry fluorescence. See Supplementary Table S3 for sgRNA library sequences.

### *In vitro* CRISPRi growth screen

HEK293T cells were transfected with the CRISPRi library along with lentiviral packaging plasmids, Delta8.2 and VSVG, and polyethylenimine at 1 mg/ml. Viral supernatants were collected on days 3 and 4 after transfection, pooled, and filtered. The A673 parental and dCas9-KRAB^+^ cell lines were transduced with the lentivirus containing the lncRNA library at a multiplicity of infection (MOI) of 0.3 with ∼1000× coverage. Once cells were transduced, they were grown for 2 days and selected with puromycin for another 4 days when cells became over 90% mCherry^+^. sgRNA-positive cells were passaged as needed for 18 days. All screens were run in two experimental duplicates. Cells were collected at day 6 post-infection (T0), after 15 doubling times (T1, 12 days post-T0) and at 22 doubling times (T2, 18 days post-T0). Cells were harvested followed by genomic DNA extraction using NucleoSpin Blood L kit, (Takara, Cat. No. 740954.20). Deep sequencing was used to determine the frequencies of sgRNAs in the plasmid library, and timepoints samples as previously described [31]. Briefly, sgRNA-containing cassettes were amplified by PCR from genomic DNA samples. During the PCR, Illumina adapters and TruSeq indexes were added, and the 270 bp PCR fragments were purified by SPRI beads. Samples were submitted for NGS Illumina sequencing with HiSeq 4000, SE50, custom sequencing primer, >500× read coverage, and 10% PhiX.

### Analysis of CRISPRi screens

The quality of fastq.gz files was evaluated using FastQC. Sequencing reads were aligned to the expected CRISPRi library sequences without tolerating any mismatches and counted. Growth scores were calculated by two different analysis pipelines, MAGeCK toolkit [32], and the Python-based ScreenProcessing pipeline (https://github.com/mhorlbeck/ScreenProcessing) [31]. To identify the lncRNAs that modify proliferation in Ewing cells, growth scores were calculated comparing all the samples and timepoints against the A673 control screen at T0. An absolute growth score of >0.8, adjusted Wald-FDR *P*-values for MAGeCK and Mann–Whitney *P*-values for ScreenProcessing were used as the cutoff for hits (see Supplementary Tables S4 and S5 for growth scores results). Out of 51 initial loci hits, 23 were prioritized by excluding those targeting pcg and those near essential pcg. The name of the nearest genes was identified via GREAT [33] and defined as “common essential” and/or “strongly selective” by published CRISPR screen data (DepMap Public 23Q4+Score, Chronos). lncRNAs with at least two sgRNAs showing significant depletion were kept and ranked by EWS expression.

### Internally controlled growth assay

To validate hits, the top two sgRNAs with the greatest effect on the screen were cloned individually in the lentivectors pLG21 (mCherry^+^ and Puro^R^) or pMB160 (GFP^+^ and Puro^R^). Internally controlled growth assays were performed by infecting A673, EW8, TC-32, TC-71, SJSA, and HEK293 dCas9-KRAB^+^ cells (constitutive or dox-inducible expression) with sgRNA lentiviruses at MOI between 0.2–0.5 and measuring the sgRNA + fraction by GFP or mCherry using flow cytometry on Accuri C6 (BD Biosciences). Three infections were created using three distinct volumes of lentivirus to serve as biological triplicates.

### Subcellular fractionation

Subcellular fractionations were carried out as described previously [34, 35]. RT-qPCR was used to determine subcellular RNA enrichment patterns by normalizing fraction Ct values to whole-cell Ct values. As fractionation quality controls, cytoplasmically enriched RNA GADPH and chromatin-enriched RNA XIST were used. All RT-qPCR primers are listed in Supplementary Table S2.

### Western blotting

Protein extracts were performed by scraping cells in RIPA buffer supplemented with protease inhibitors (Roche) on ice. Protein quantitation was determined by BCA assay (Thermo Fisher, Cat. No. 23225) and run on 4%–15% gradient gels (Bio-Rad TGX) followed by transfer to PVDF membrane using a semi-dry system (Bio-Rad Trans-Blot Turbo Transfer System). Immunoblotting was performed overnight at 4°C with the following primary antibodies at the indicated dilutions: cleaved PARP (Cell Signaling, Cat. No. 5625, at 1:1000), SLC3A2 (Cell Signaling, Cat. No.13180, at 1:1000), and α/β-Tubulin (Cell Signaling, Cat. No. 2148, at 1:1000). Bio-Rad Clarity ECL kit was used for chemiluminescence detection, and the ChemiDoc XRS System (Bio-Rad) was utilized for imaging. Western quantitation was performed using Image Lab software (Bio-Rad).

### Internally controlled growth assay in tumor xenografts

A673 dCas9-KRAB cells were transduced with lentivirus vectors for the indicated sgRNA. Cells were selected with puromycin, and then stable cell lines were expanded. On the day of the xenograft implantation (Day 1), cells expressing non-targeting sgRNA (mCherry^+^) were pooled 1:1 with non-targeting sgRNA (GFP^+^), or SNHG1-targeting sgRNA (GFP^+^), SNHG12-targeting sgRNA (GFP^+^), or SNHG30-targeting sgRNA (GFP^+^). Four different pooled populations (GFP^+^/mCherry^+^) were resuspended in a mixture of serum-free DMEM (Corning, Cat. No.15-017-CV) and Matrigel (Corning, Cat. No.356237). Pooled mixes were injected subcutaneously into both flanks of NSG mice (2 × 10^6^ cells per flank, three or four mice per condition) and tumors were allowed to develop for 30 days. Then mice were sacrificed, and tumor were dissected, chopped, and dissociated into single-cell suspension using phosphate buffered saline (PBS) supplemented with Collagenase/Dispase (Roche, Cat. No.11097113001) and DNAse I (Worthington, Cat. No.LS002006) at 37°C for 30 min with agitation. After passing through a 40 µm filter, digested samples were centrifuged and resuspended in complete DMEM medium. Cell pellets were treated for 1 min in RBC buffer (155 mM NH_4_Cl, 12 mM NaHCO_3_, 0.1 mM ethylenediaminetetraacetic acid), and then resuspended in complete DMEM and centrifuged to remove red blood cells. Cell pellets were reconstituted in PBS containing 1% serum and flow cytometry was performed on an SH800S Cell Sorter (Sony Biotechnology). The ratio of GFP to mCherry fluorescence in tumor samples was normalized to Day 1 of cell populations and reported as a fold-change relative to the non-targeting controls (sgNT GFP/sgNT mCherry).

### Rescue experiment with SNHG1 overexpression using Sleeping Beauty transposon system

Plasmids expressing cDNA SNHG1 (pTD2) or the complete genomic SNHG1 sequence (pTD4) were co-transfected with pTS395_PhCMV-SB100 into A673 cell line using Lipofectamine 3000 (Thermo Fisher Cat. No L3000015). After puromycin selection, these cells were transduced with a low titer of lentivirus sgNT or sgSNHG1 (GFP^+^). The sgSNHG1 targeted the 5′UTR of the endogenous SNHG1 gene, therefore, the extra SNHG1 copies from transposon integration were not affected by the sgSNHG1. The result was a mixed population of dTomato^+^ (SNHG1), GFP^+^ (sgRNA^+^), or GFP^+^/dTomato^+^ cells (yellow). We sorted the yellow population to determine the expression of SNHG1 by RT-qPCR, and we used the mixed population to perform a internally controlled growth assay tracking yellow cells by flow cytometry.

### RNA-seq

Flash-frozen patient tumors were processed for RNA-seq as previously described [36]. RNA-seq of EWS502 N-dTAG EWS–FLI1 cell line was carried out as previously described [27]. Dox-inducible SNHG knockdown cells, Ewing commercial cell lines, and PDXC cell lines were also processed for RNA-seq. In each instance, cells were scraped, harvested, and total RNA was isolated using QIAshredder homogenization (Qiagen, Cat. No.79654) and RNAeasy column kit (Qiagen, Cat. No.74106). RNA was quantified with a NanoDrop 2000c (Thermo Fisher) and RNA integrity was analyzed using High Sensitivity RNA ScreenTape (Agilent, Cat. No.5067-5579) on a TapeStation 4200 (Agilent). The efficiency of the SNHG knockdowns was performed by RT-qPCR before submitting for RNA-seq.

RNA-seq libraries were created using the TruSeq Stranded mRNA Kit (Illumina, Cat. No.RS-122-2101) as directed by the manufacturer. All manufacturer controls were used in the library preparation. The High Sensitivity D1000 ScreenTape (Agilent, Cat. No.5067-5584) was used to quantify libraries on the TapeStation 4200 (Agilent). The sequencing was performed at the Center for Advanced Technology (UCSF) using Illumina NovaSeq 6000 machine with 150 bp paired-end reads chemistry, with raw FASTQ files provided directly by the facility. RNA-seq data for the dox-inducible SNHG knockdown cells were processed as follows: adapters and low-quality regions were trimmed using the Trimmomatic tool (v0.36). The processed reads were then mapped to the human reference genome (hg38) using the STAR aligner (v2.5.1b). To quantify gene-level expression, we utilized the STAR aligner’s “quantMode” feature with gene annotations sourced from GENCODE p5. Quality control metrics, including counts of uniquely aligned reads, ratios of unique-to-multiple alignments, and exon-to-intron ratios, were evaluated using ngsutilsj (v0.3-2180ca6). Further QC assessments and subsequent analyses were all completed with R (v3.5.3). Aligned reads were normalized using the trimmed mean of *M*-values (TMM) method from the EdgeR package (v3.24.3), and counts were transformed as log_2_(cpm + 1). To detect statistically significant alterations in gene expression, the VOOM function from the limma package was utilized to estimate the mean–variance relationship and assign precision weights. These were subsequently integrated into the empirical Bayes linear modeling framework provided by limma (v3.38.3) to calculate statistical outputs, including *P*-values, adjusted *P*-values, and log-fold changes (LogFC).

### Pathway analysis

We conducted Gene Set Enrichment Analysis (GSEA) in SNHG1, SNHG12, and SNHG30 knockdown cells using the R package fgsea on pre-ranked LogFC values (https://github.com/ctlab/fgsea/). This approach is based on the method proposed by Subramanian *et al.* in 2005 [37]. The predefined gene sets for GO, Reactome, Hallmark, and KEGG pathways were acquired from the Molecular Signatures Database (MSigDB).

### RiboMethSeq and HydraPsiSeq

Quantification of 2′-O-methylation (Nm) and pseudouridylation (Ψ) of rRNAs was performed by RiboMethSeq and HydraPsiSeq, as previously described [38, 39]. Briefly, for RiboMethSeq, total RNA was subjected to alkaline hydrolysis for 14 min at 96°C followed by end-repair and library preparation using NEBNext^®^ Small RNA Library kit (NEB, USA). Libraries were multiplexed and sequenced on Illumina NextSeq2000 in single read 50 nt mode (SR50). Details of bioinformatic analysis are described in Marchand *et al.* [39]. We determined the 2′-O-methylation levels for previously reported 67 positions in 28S rRNA, 41 sites in 18S rRNA, and 2 positions in 5.8S rRNA [40]. Methylation Score (MethScore) was calculated for each position to assess the fraction of rRNA molecules modified at a given position in A673 control and knockdown cells. The affected sites between sgNT versus SNHG1 KD, sgNT versus SNHG12 KD, sgNT versus SNHG30 KD, and shGFP versus shFLI1 KD were defined as a difference of absolute 0.08 in the MethScore, and a threshold of adjusted *P*-value <.05.

For HydraPsiSeq analysis, 200 ng of total RNA was subjected to hydrazine treatment (50% final concentration) for 45 min on ice followed by ethanol precipitation. The pellet was resuspended in 1 M aniline, pH 4.5, and incubated for 15 min at 60°C in dark and precipitated in ethanol. RNA was then 3′-end dephosphorylated using T4 PNK in 100 mM Tris–HCl, pH6.5, 100 mM MgOAc, and 5 mM β-mercaptoethanol and incubated for 6 h at 37°C. After enzyme inactivation, RNA was extracted by phenol:chloroform and ethanol precipitated. RNA was then converted to library using NEBNext^®^ Small RNA Library kit (NEB, USA). Libraries were multiplexed and sequenced on Illumina NextSeq2000 in single read 50 nt mode (SR50). Details of bioinformatic analysis are described in Pichot *et al.* and Marchand *et al.* [41, 42]. See Supplementary Tables S9–S11 for RiboMethSeq and HydraPsiSeq Raw data.

### Data acquisition

TARGET (from Genomic Data Commons Data Portal) TPM data were downloaded from the UCSC Treehouse Public Data repository (https://treehousegenomics.soe.ucsc.edu/public-data/). For comparative analyses with this dataset, we additionally reprocessed our RNA-seq data with the UCSC Toil RNA-seq pipeline (https://github.com/BD2KGenomics/toil-rnaseq) to ensure harmonization of the two datasets for direct cross-dataset comparisons. The cancer cell lines’ genetic dependencies for SLC3A2 estimated from CRISPR screen using Chronos algorithm (DepMap Public 26Q1+Score, Chronos) [43] were downloaded from the DepMap website (https://depmap.org/portal/).

Sequence similarity between humans and other vertebrates for SNHG genes was analyzed with the ECR Browser [44] (https://ecrbrowser.dcode.org/) based on sharing at least 70% sequence identity across 100-base-pair ECR length.

### Statistics

Statistical analysis was conducted using GraphPad Prism 7 or with R. Statistical parameters such as *P*-value, adjusted *P*-value, SEM, SD, and FDR are described on the figures and in the appropriate figure legends.

## Results

### Identification of candidate lncRNA for evaluation in Ewing sarcoma

**Figure 1.**
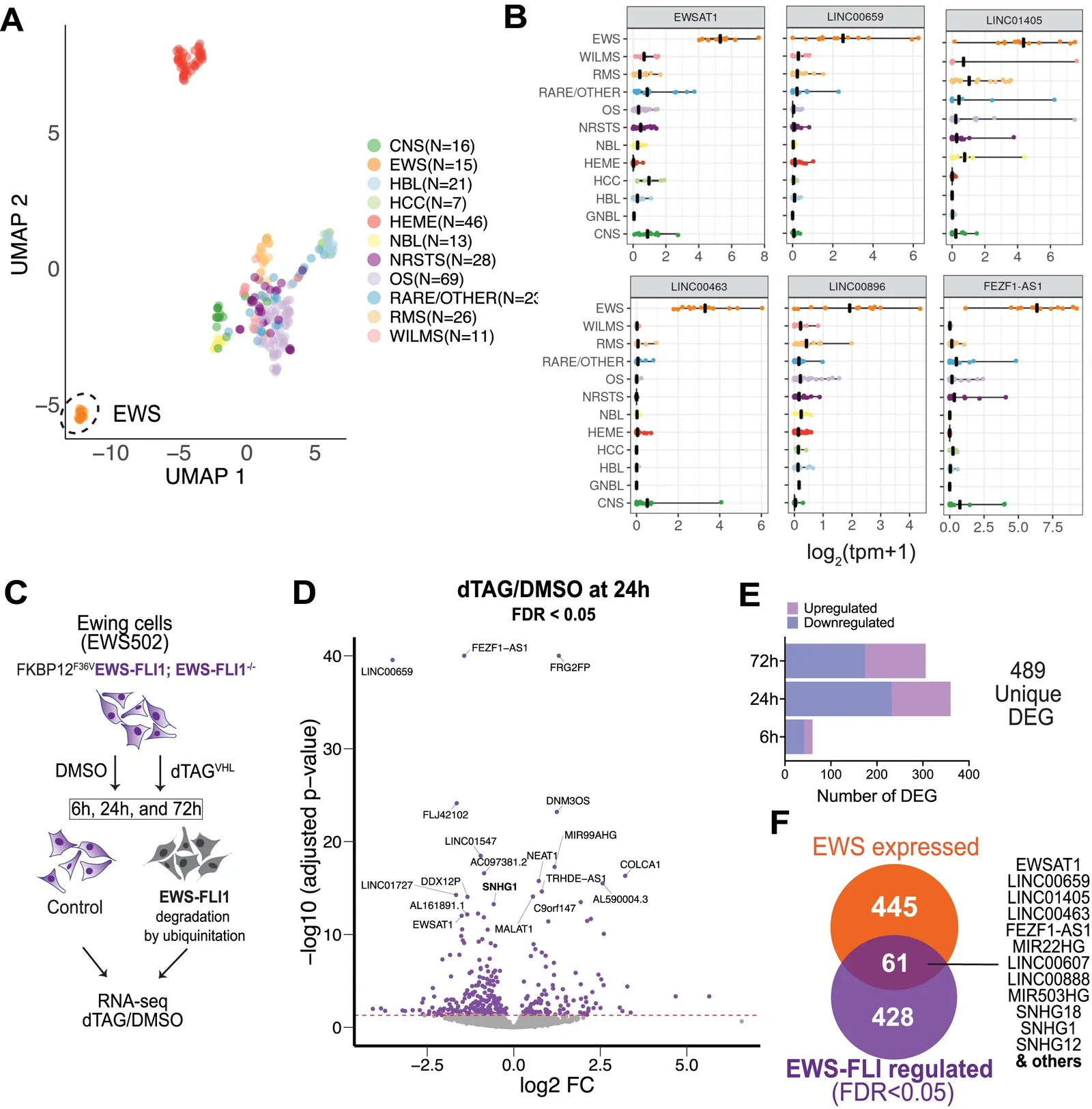
LncRNA signature in EWS. (**A**) UMAP showing clustering of RNA-seq profiles of a large collection of pediatric cancers based on the expression of lncRNAs. (**B**) Expression of selected lncRNA genes in EWS compared to other indicated cancer types. (**C**) Scheme of inducible EWS–FLI1 degradation system in EWS502 cells for RNA-seq. (**D**) Volcano plot of lncRNA genes differentially expressed in EWS502 FKBP12^F36V^-EWS–FLI1 cells treated with either DMSO or 1 μM of dTAG^VHL^ for 24 h. Purple dots represent ncRNA genes with FDR < 0.05; −log₁₀(padj) values are capped at 40 for visualization. (**E**) Number of down- and up-regulated ncRNA genes in EWS502 FKBP12^F36V^-EWS–FLI1 cells at three different timepoints and the total of DEGs (union of DEG at 6, 24, and 72 h after removal of duplicates). (**F**) Venn diagram displaying overlap between lncRNAs expressed in primary Ewing tumors versus ncRNAs regulated by EWS–FLI1 (DEGs from dTAG^VHL^ treatment for 6, 24, and 72 h in EWS502 FKBP12^F36V^-EWS–FLI1 cells). CNS, central nervous system tumors; EWS, Ewing sarcoma; HBL, hepatoblastoma; HCC, hepatocellular carcinoma; HEME, hematologic (referring to blood cancers or disorders); NBL, neuroblastoma; NRSTS, non-rhabdomyosarcoma soft tissue sarcoma; OS, osteosarcoma; RARE/OTHER, rare or other tumors; RMS, rhabdomyosarcoma; WILMS, Wilms tumor; GNBL, ganglioneuroblastomas.

We first sought to systematically identify lncRNAs consistently expressed in EWS and upregulated compared to other pediatric cancers. We evaluated lncRNA expression in an RNA-seq dataset of pediatric cancers from 11 subtypes including 15 EWS. Applying Uniform Manifold Approximation and Projection (UMAP) for dimensionality reduction with only lncRNA transcript data demonstrated clustering and clear separation of hematologic malignancy and EWS from other pediatric tumors (Fig. 1A). A number of lncRNAs including LINC00896, FEZF1-AS1, LINC01405, LINC00463, LINC00659, and EWSAT1 are highly and in some cases uniquely expressed in EWS (Fig. 1B and Supplementary Fig. S1A).

Expression of lncRNAs in EWS could be a consequence of the cell of origin or due to direct regulation by EWS–FLI1. To distinguish these two possibilities, we deployed a degradation tag (dTAG^VHL^) to induce rapid degradation of EWS–FLI1 [45]. Ewing cell line EWS502 expressing N-dTAG EWS–FLI1 was treated with either DMSO or 1 μM dTAG^VHL^ (Fig. 1C and Supplementary Fig. S1B and C). At 6, 24, and 72 h timepoints, RNA was collected and used for RNAseq (Fig. 1D and Supplementary Fig. S1D and E). Degradation of EWS–FLI1 led to changes in expression of 489 unique ncRNAs (including lncRNAs, pseudogenes, and small RNAs) across 6, 24, and 72 h (FDR < 0.05; Fig. 1E and Supplementary Table S6). Of these, 290 were downregulated and 200 were upregulated at one or more timepoints, with one gene meeting significance in opposite directions at different timepoints (MIR3648-1).

Sixty-one lncRNAs were identified as expressed in EWS primary samples and also significantly regulated by EWS–FLI1 (Fig. 1F). Among them, EWSAT1, LINC00659, LINC01405, and LINC00463 were also among the top lncRNA expressed in EWS (Fig. 1B and Supplementary Fig. S1A). This confirmed previously published data that EWSAT1 is regulated by EWS–FLI1 [14]. Taken together our results suggest that EWS has a distinct lncRNA signature compared to other pediatric cancers. Some of these lncRNAs are regulated by EWS–FLI1, whereas others are highly expressed in these tumors due to their cell of origin.

### CRISPRi-based screen identifies functional lncRNAs in Ewing sarcoma

**Figure 2.**
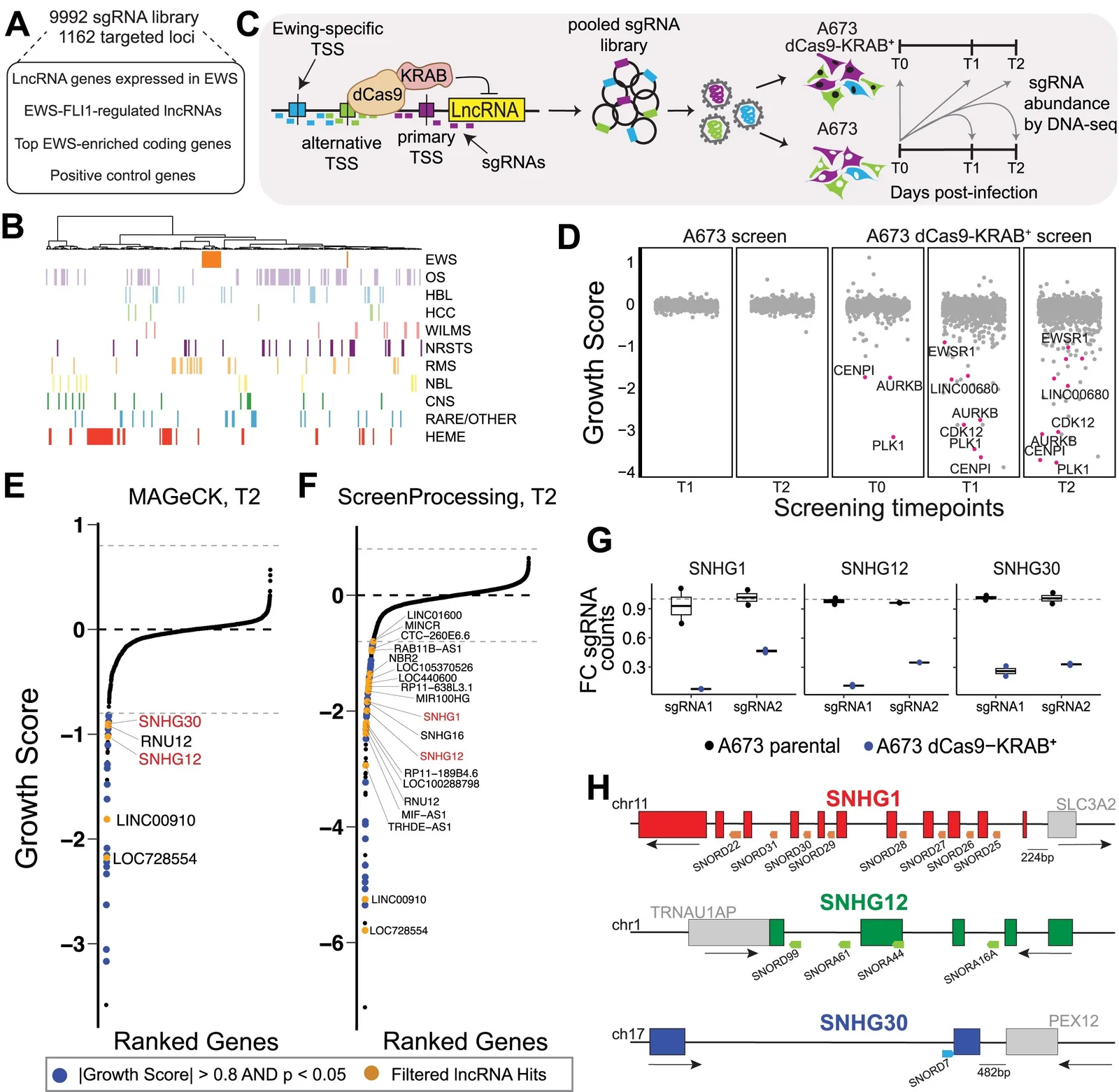
CRISPRi screen reveals lncRNAs dependencies in EWS. (**A**) Schematic for design of the Ewing-custom CRISPRi pooled library. (**B**) Cluster analysis of gene expression for genes included in the CRISPRi library in primary tumors for pediatric cancers. Tumor types are abbreviated as in Fig. 1. (**C**) Schematic of Ewing-specific TSS prediction, pooled sgRNA library design, and workflow for *in vitro* CRISPRi growth screen in A673 parental (control) and A673 dCas9-KRAB+ cells. Cells were harvested at T0, T1, and T2 for quantification of sgRNA abundance. (**D**) Growth scores for both A673 parental (control) and A673 dCas9–KRAB+ cells at different timepoints (T0, T1, and T2) analyzed by MAGeCK. Only positive control genes with significant effects (|Growth Score| > 0.8 and FDR < 0.01) are shown in pink; selected genes are labeled. (**E**) Growth screen results at timepoint 2 (T2) analyzed using MAGeCK. (**F**) Growth screen results from timepoint 2 (T2) analyzed using ScreenProcessing. Genes with significant effects (|Growth Score| > 0.8 and *P* < .05) are indicated, with key filtered lncRNA hits highlighted separately as shown in the panel legend (prioritized as described in Supplementary Fig. S3D). Statistical significance was determined using adjusted Wald-FDR *P*-values for MAGeCK and Mann–Whitney *P*-values for ScreenProcessing. (**G**) Fold change of read counts for two sgRNAs targeting SNHG1, SNHG12, and SNHG30. A673 parental screen and A673 dCas9-KRAB^+^ cells are indicated as shown in the panel legend. Two independent replicates shown. (**H**) Schematic representations of SNHG1, SNHG12, and SNHG30 loci. Each gene structure shows intron–exon organization, intronic snoRNAs genes, and neighboring pcg in gray. Distances between SNHG genes and neighboring genes are indicated.

To systematically identify lncRNAs that modify proliferation in EWS, we designed an sgRNA library to enable a high-throughput CRISPRi screening approach [31]. We included in this library sgRNAs targeting (i) lncRNA genes expressed in EWS tumor samples, (ii) lncRNAs regulated by EWS–FLI1, (iii) a subset of pcg that are highly and specifically expressed in EWS, and (iv) positive control genes known to be required for proliferation [31]. The pooled CRISPRi sgRNA library contained a total of 9992 sgRNAs targeting 2212 TSSs across 1162 loci and includes 805 non-targeting sgRNAs (Fig. 2A). Analysis of RNA expression in tumor samples using this list of genes again demonstrated that only EWS tumor samples cluster tightly, supporting that this is a unique set of EWS-expressed ncRNAs (Fig. 2B and Supplementary Fig. S2A). As lncRNAs genes can have multiple TSSs, which may be tissue- or tumor-type specific [29, 46, 47], we designed sgRNAs for the CRISPRi library to target all possible TSSs including canonical, alternative, and Ewing-specific TSS (Fig. 2C and Supplementary Fig. S2B and C).

We generated a set of stable EWS–FLI1 expressing cell lines expressing dCas9-KRAB (Supplementary Fig. S2D) and chose A673 for the initial CRISPRi screen. Parental and dCas9-KRAB^+^ A673 cells were transduced with lentivirus containing the lncRNA sgRNA library. Cells were collected and genomic DNA was processed for sequencing at T0, after 15 doubling times (T1), and at 22 doubling times (T2) (Fig. 2C). The abundance of each sgRNA in the lncRNA EWS library was quantified by sequencing. Growth scores were calculated using two different analysis pipelines (see the “Materials and methods” section). All the samples and timepoints were compared to the control screen at T0 (Fig. 2C).

We detected drop out of specific sgRNAs in the A673 dCas9-KRAB cells but not in parental cells (Fig. 2D). Among the positive control genes included in the library, 55% (16 of 29) scored as negatively selected, confirming the ability of this library to identify strong negative selection. Positive controls included EWSR1 and CDK12 (Fig. 2D). Negative selection for sgRNAs targeting EWSR1 would be expected as it would lead to downregulation of the driving oncogenic event EWS–FLI1. Similarly, inhibition of CDK12 has previously been shown to be strongly inhibitory in Ewing cells as it leads to the downregulation of DNA repair genes [48]. We identified 51 negatively selected loci (hits) by combining results from both analysis pipelines across timepoints and excluding positive controls. No positively selected loci were detected (see the “Materials and methods” section; Fig. 2E and F, Supplementary Fig. S3A–D, and Supplementary Tables S4 and S5). To select hits for subsequent analysis, we filtered the 51 initial loci hits by excluding pcg hits and those neighboring essential coding genes (Supplementary Fig. S3D and Supplementary Table S7). We kept 11 loci hits targeting lncRNAs with at least two depleted sgRNAs and ranked them based on expression levels in primary EWS tumors. Three of the most expressed filtered lncRNA hits belong to the family of SNHG (Supplementary Fig. S3E). SNHG1, SNHG12, and SNHG30 were chosen for further evaluation (Fig. 2G and H).

Of the 51 negatively selected hits, 19 are significantly regulated by EWS–FLI1 (FDR < 0.05; Supplementary Fig. S3F). Notably, SNHG1 and SNHG12 were also identified as EWS–FLI1-regulated genes (Supplementary Fig. S3F). SNHG1 is significantly downregulated at all three timepoints (6 h: log_2_FC −0.20, padj 1.8 ×10^-3^; 24 h: log_2_FC −0.58, padj 5.6 ×10^-14^; 72 h: log_2_FC −0.83, padj 2.1 ×10^-33^), and is present in the published EWS–FLI1 consensus signature [12], supporting direct transcriptional regulation. SNHG12 reaches significance at 24 and 72 h (log_2_FC −0.52, padj 0.010; log_2_FC −0.49, padj 0.030), suggesting delayed or indirect regulation. SNHG30 is not differentially expressed at any timepoint. Notably, SNORD99, an intronic snoRNA of SNHG12, was also downregulated at 24 h (log_2_FC −2.61, padj 0.0008), likely through indirect regulation (Supplementary Fig. S4A–C and Supplementary Table S6).

### Loss of SNHG genes decreases proliferation, increases cell death, and decreases tumor growth *in vivo*

**Figure 3.**
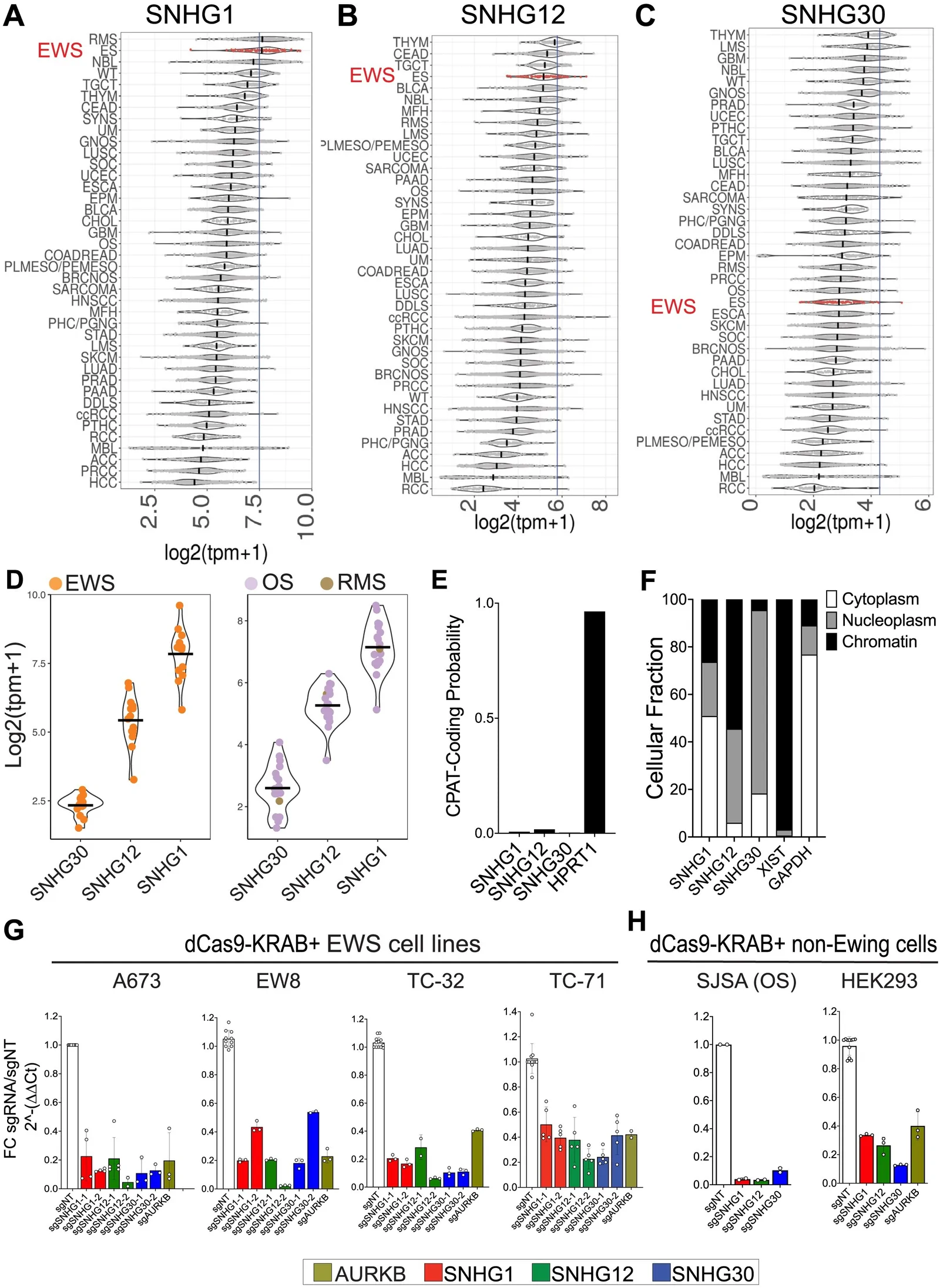
SNHG genes in EWS. (**A**) SNHG1 expression across pediatric and adult tumor samples from the UCSC Treehouse Public Data collection (see the “Materials and methods” section). (**B**) SNHG12 expression in UCSC Treehouse. (**C**) SNHG30 expression in UCSC Treehouse. Cancer type abbreviations used in panels (A)–(C) are defined in Supplementary Table S8. (**D**) Expression of SNHG1, SNHG12, and SNHG30 in EWS, osteosarcoma (OS), and rhabdomyosarcoma (RMS) cell lines from RNA-seq data. Three PDXC cell lines PID285, PID290, and PID799 are included for EWS. (**E**) Coding potential of SNHG1, SNHG12, and SNHG30 genes called by CPAT. HPRT1 is used as positive control for coding potential. (**F**) RNA levels of SNHG1, SNHG12, and SNHG30 in A673 dCas9-KRAB^+^ cells following subcellular fractionation. XIST and GAPDH used as controls for the chromatin and cytoplasmic fractions, respectively. (**G**) RT-qPCR analysis of SNHG1, SNHG12, SNHG30, and AURKB expression in EWS cell lines (A673, TC-71, TC-32, and EW8 dCas9-KRAB^+^) and (**H**) non-Ewing cell lines (SJSA and HEK293 dCas9-KRAB^+^) transduced with indicated sgRNAs. (*n* = 2–4). Relative transcript levels in RT-qPCRs were normalized to HPRT1 then fold change sgRNA/sgNT was calculated. Error bars represent mean ± SD.

SNHG1 encodes eight snoRNAs (SNORD22, SNORD25, SNORD26, SNORD27, SNORD28, SNORD29, SNORD30, and SNORD31) processed from its spliced introns [21], SNHG12 codes four snoRNAs (SNORA44, SNORA61, SNORA16A, and SNORD99) and SNHG30 encodes one intronic snoRNA (SNORD7) (Fig. 2H). Some SNHG genes, including SNHG1 and SNHG12, have been reported to be dysregulated in cancer [49, 50]. Notably, SNHG1 and SNHG12 are highly expressed in Ewing samples compared with other cancers (Fig. 3A and B), while SNHG30 exhibits average expression across all tumors examined (Fig. 3C; see Supplementary Table S8 for definitions of disease abbreviations). A similar trend was observed in cell lines for EWS, osteosarcoma (OS), and rhabdomyosarcoma (RMS), where SNHG1 and SNHG12 are more highly expressed than SNHG30 (Fig. 3D).

SNHG1, SNHG12, and SNHG30 were confirmed as ncRNAs using the CPAT algorithm [51] (Fig. 3E). In Ewing cells, SNHG12 and SNHG30 RNAs primarily reside in the nucleus, whereas SNHG1 is found both in the nucleus and the cytoplasm (Fig. 3F) [52, 53]. We evaluated the functional consequences of the loss of these SNHG genes in EWS by performing internally controlled growth assays. Two sgRNAs for each SNHG were cloned into a lentiviral vector containing a GFP expression cassette. These lentiviruses were used to infect four dCas9-KRAB expressing EWS cell lines: A673, EW8, TC-32, and TC-71. Knockdown of the corresponding SNHG RNAs was confirmed by RT-qPCR (Fig. 3G). To determine whether loss of these lncRNAs is a specific vulnerability for EWS cells, we infected two non-EWS cell lines, SJSA (osteosarcoma) and HEK293, and confirmed knockdown by RT-qPCR (Fig. 3H).

**Figure 4.**
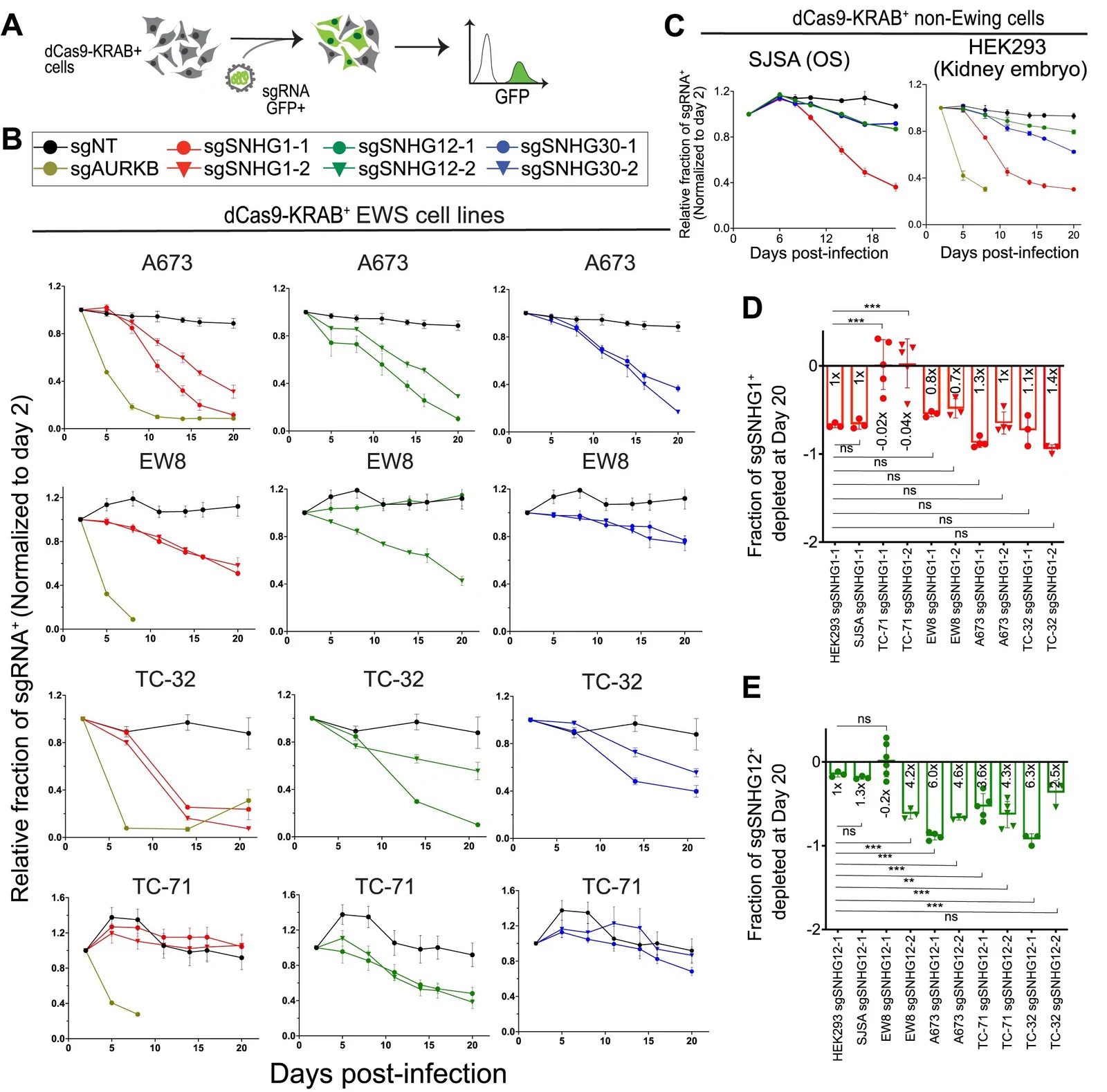
SNHG1, SNHG12, and SNHG30 genes are required for cellular proliferation in EWS. (**A**) Scheme of internally controlled growth assays: cells expressing dCas9-KRAB were infected with lentiviral sgRNA vectors containing GFP. sgRNA+ populations were monitored over time by collecting GFP+ cells with flow cytometry. (**B**) Internally controlled growth assays performed in the indicated dCas9-KRAB^+^ Ewing cell lines stably expressing non-targeting sgRNA (sgNT), two independent sgRNAs targeting SNHG genes. sgAURKB used as positive control. Plotted is the relative sgRNA+ population normalized to the day 2 after infection. Data represent mean ± SEM, *n* = 3–7. (**C**) Internally controlled growth assays performed in two dCas9-KRAB^+^ non-Ewing cells stably expressing the indicated sgRNA data represent mean ± SEM, *n* = 3–8. (**D**) Negative values indicate the fraction of sgSNHG1^+^ cells depleted at day 20 [from panels (B) and (C)] for each dCas9-KRAB^+^ cell line. Each bar plot is labeled with the specific fold change of sgSNHG1^+^ depletion versus HEK293. Data represent the mean ± SD. One-way ANOVA followed by Dunnett’s multiple comparison test where “ns” indicates nonsignificant (*P*-value >.05); * denotes *P*-value ≤.05, ** denotes *P*-value ≤.01, *** denotes *P*-value ≤.001. (**E**) Fraction of depleted SNHG12^+^ cells as described in panel (D).

The sgRNA+ populations were monitored by measuring the fraction of GFP+ cells by flow cytometry over time (Fig. 4A and Supplementary Figs S5–S9). Ewing cells transduced with sgRNAs targeting SNHG1, SNHG12, or SNHG30 were depleted relative to uninfected cells. In contrast, cells expressing a non-targeting sgRNA (sgNT) were maintained (Fig. 4B). An sgRNA targeting AURKB was used as a positive control. SNHG1 showed a significant decrease in GFP^+^ cells after day 10 post-infection. SNHG1 KD cells were also depleted in both non-EWS cell lines (Fig. 4C and Supplementary Fig. S9A and B), indicating that SNHG1 represents a pan-essential gene required for cell fitness rather than an EWS-specific dependency (Fig. 4D). Depletion of SNHG30 KD cells was most pronounced in the EWS cell lines A673 and TC-32 when comparing with other EWS and non-EWS cells (Fig. 4B and C, and Supplementary Figs S5, S7, S9A and B, and S10A). In contrast, SNHG12 KD led to significantly greater depletion in EWS lines compared to SJSA and HEK293 (Fig. 4E and Supplementary Figs S5–S8 versus S9A and B), suggesting an enhanced vulnerability to SNHG12 loss in EWS cells.

**Figure 5.**
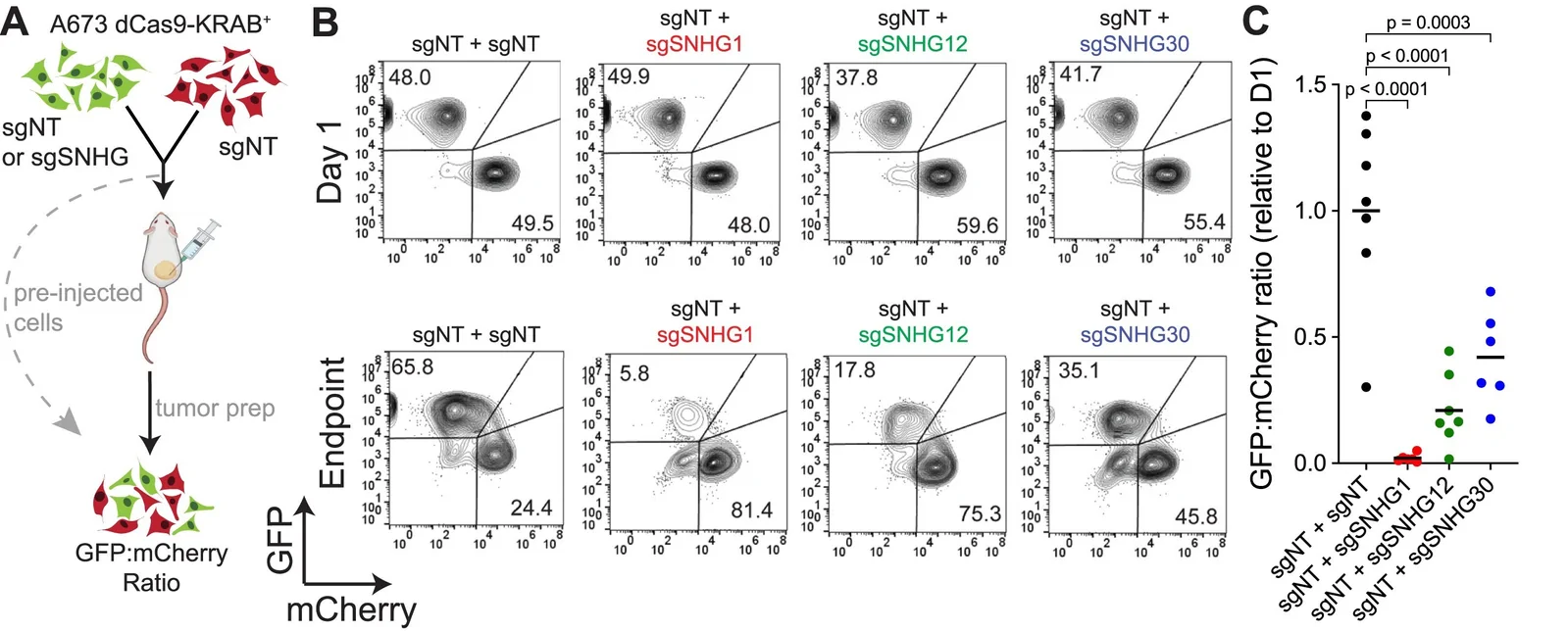
*In vivo* role of SNHG1, SNHG12, and SNHG30. (**A**) Scheme for the *in vivo* internally controlled growth assay in A673 xenografts. (**B**) Flow cytometry of day 1 A673 cell populations (top) compared to representative endpoint tumor cell populations (bottom). (**C**) Fold change in GFP:mCherry ratios between A673 xenograft tumor cell populations at endpoint and day 1. Data normalized against sgNT + sgNT control. *n* = 7 individual tumors assessed for “sgNT + sgNT,” and “sgNT + sgSNHG12.” *n* = 6 individual tumors assessed for “sgNT + sgSNHG1,” and “sgNT + sgSNHG30.” Lines represent means. ANOVA followed by Dunnett’s test between indicated sgRNA mix and “sgNT + sgNT” control.

Depletion in cell competition may be due to apoptosis or senescence [54–56]. Higher levels of cleaved PARP protein were detected in SNHG KD genes compared to sgNT in A673 cells (Supplementary Fig. S10B and C), but not other EWS lines (Supplementary Fig. S10D–F) [57], suggesting that cell loss in this system is driven primarily by a proliferative disadvantage rather than acute apoptosis. SNHG1, SNHG12, and SNHG30 dependency in Ewing cells was further tested using an *in vivo* internally controlled growth assay (Fig. 5A). A673 cells expressing a GFP-linked SNHG sgRNA were depleted in tumor xenografts in NSG mice, as indicated by a significant decrease in the GFP^+^ proportion of engrafted cells. In contrast, cells that expressed a non-targeting sgRNA were preserved (Fig. 5B and C). These findings support a role for lncRNAs SNHG1, SNHG12, and SNHG30 in the EWS cell fitness.

### Transcriptional effects of SNHG1, SNHG12, and SNHG30 loss in EWS

**Figure 6.**
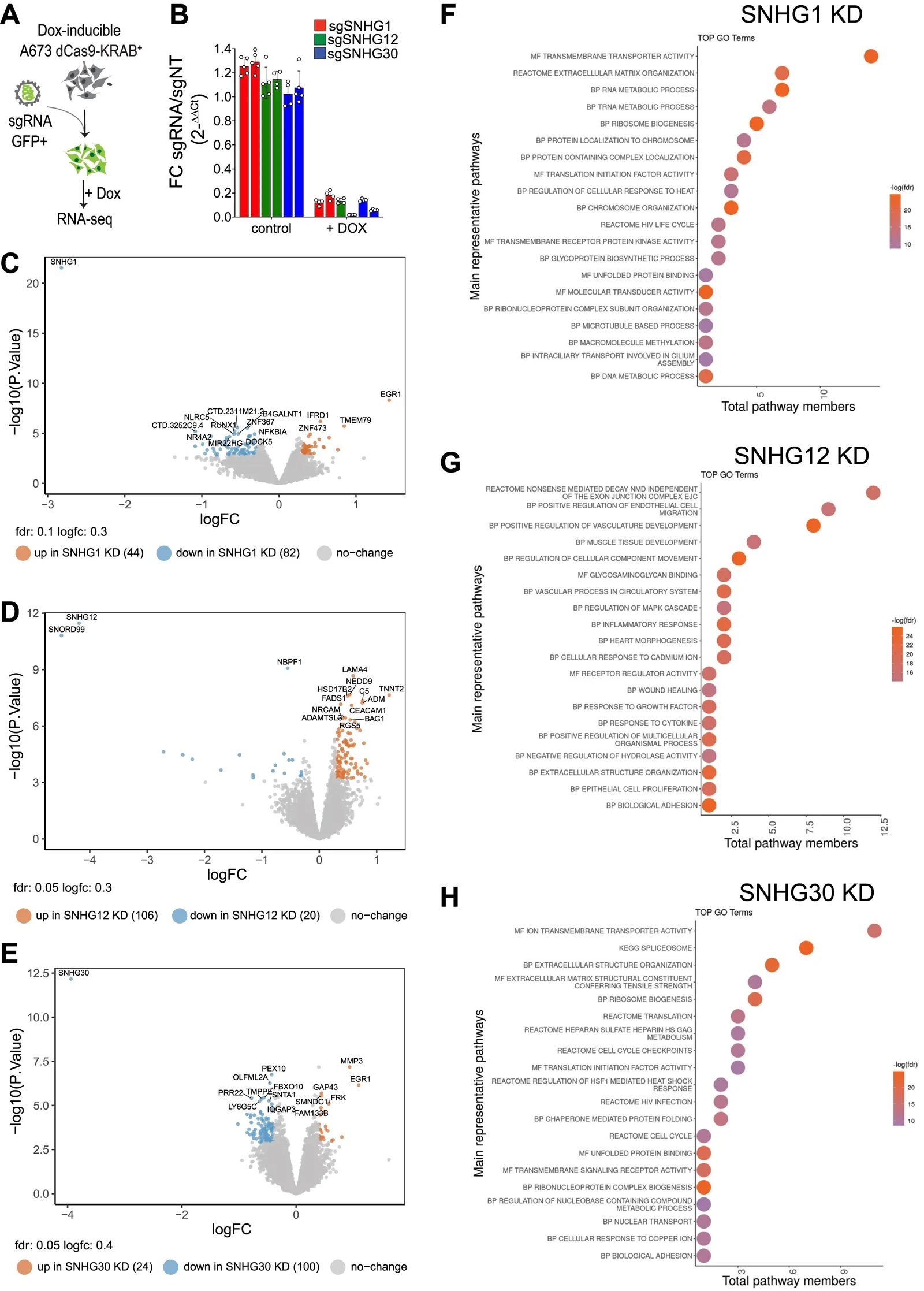
RNA-seq analysis of SNHG knockdowns. (**A**) Schema for the RNA-seq experiment. Cells were harvested for RNA after 6 days of induction with doxycycline. (**B**) RT-qPCR analysis of SNHG1, SNHG12, and SNHG30 expression in A673 dox-inducible dCas9-KRAB^+^ cells transduced with indicated sgRNAs. Cells were harvested after 6 days post-induction with doxycycline. Relative ncRNAs levels were normalized to HPRT1 levels then fold change sgRNA/sgNT was calculated. Data represent mean ± SD (*n* = 3–6). (**C**) Volcano plot of differential gene expression after 6 days of SNHG1 knockdown. (**D**) Volcano plot of differential gene expression after 6 days of SNHG12 KD. (**E**) Volcano plot of differential gene expression after 6 days of SNHG30 KD. (**F**–**H**) Gene Set Enrichment Analysis (GSEA) using KEGG, GO terms, and Reactome gene sets on differentially expressed ranked genes between SNHG knockdowns and control cells. Plots show the representative pathway with lowest *P*-value and the total number of aggregated pathway members.

RNA-seq was used to identify transcription-dependent mechanisms underpinning the observed effects of SNHG loss. Parental A673 cells were compared to those with knockdown for SNHG1, SNHG12, or SNHG30 (Fig. 6A and B). For SNHG1, 44 upregulated and 82 downregulated genes were identified (Fig. 6C). For SNHG12, 106 upregulated and 20 downregulated genes were identified (Fig. 6D).

For SNHG30, 24 genes were upregulated and 100 genes were downregulated (Fig. 6E). For all three knockdowns the most significantly downregulated gene was the corresponding SNHG, demonstrating specificity of the sgRNA used in each case (Fig. 6C–E).

We performed Gene Set Enrichment Analysis (GSEA) and aggregated all pathways into related enriched groups (Fig. 6F–H). SNHG1 KD led to significant enrichment of “RNA metabolic process” (*P*-value <1 × 10^−7^) and “Ribosome biogenesis” (*P*-value <1 × 10^−7^) (Fig. 6F and Supplementary Fig. S11). SNHG12 KD regulated a distinct set of pathways including “Nonsense-mediated decay NMD independent of the exon junction complex” (*P*-value = 5.9 × 10^−6^), “Positive regulation of endothelial cell migration” (*P*-value = 2.4 × 10^−5^), and “Positive regulation of vasculature development” (*P*-value <1 × 10^−7^) (Fig. 6G and Supplementary Fig. S12). For SNHG30 KD, GSEA revealed pathway enrichment for “Ion transmembrane transporter activity” (*P*-value = 1.4 × 10^−5^), “Spliceosome” (*P*-value < 1 × 10^−7^), “Extracellular structure organization” (*P*-value = 2 × 10^−7^), and “Ribosome biogenesis” (*P*-value = 1.4 × 10^−6^) (Fig. 6H and Supplementary Fig. S13). These results support distinct transcriptional consequences of loss of expression of each SNHG in EWS.

### Rescue of the slow proliferation phenotype in SNHG1 KD cells

**Figure 7.**
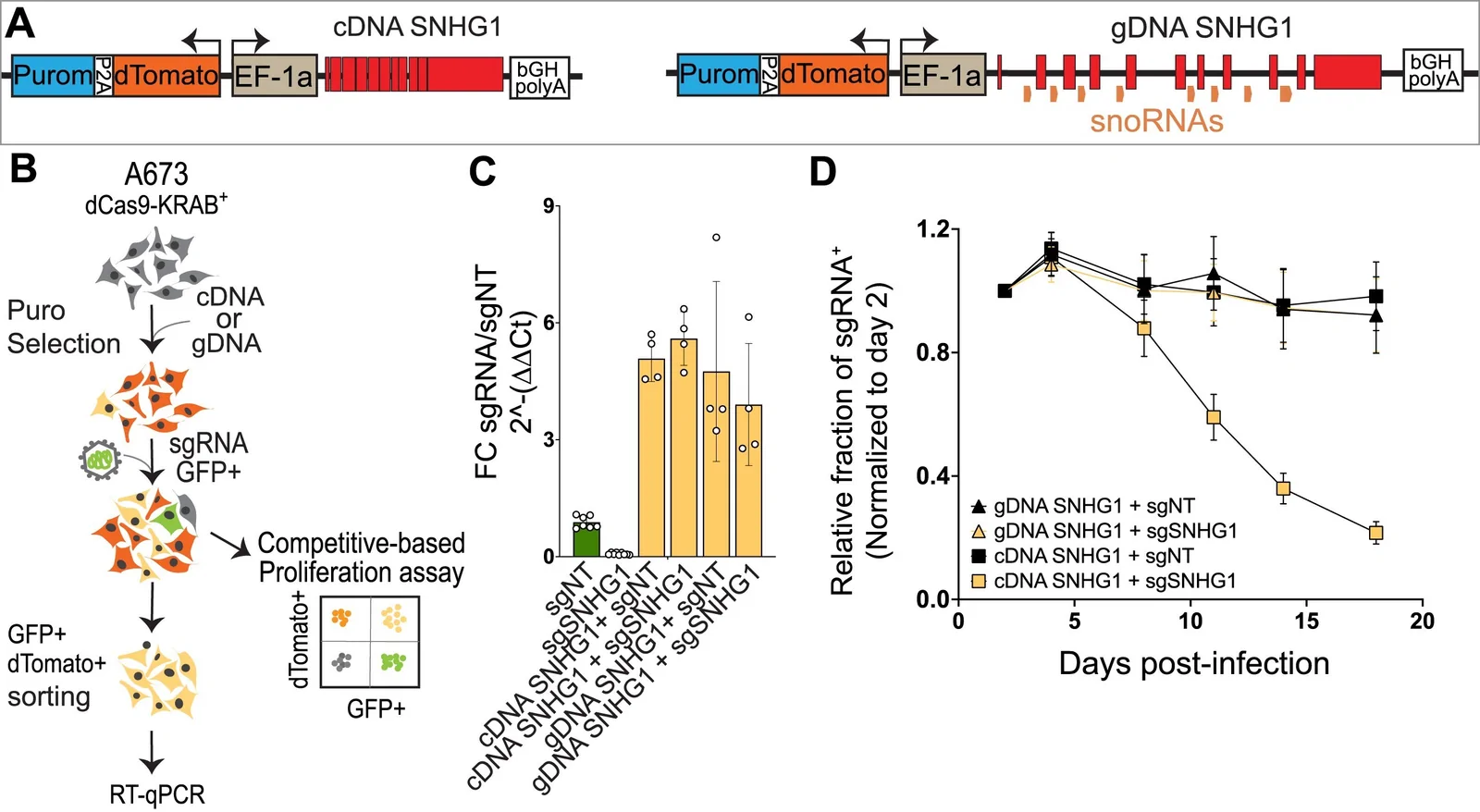
Rescue of proliferation phenotype in SNHG1 KD. (**A**) Scheme for Sleeping Beauty transposon constructs expressing extra copies of SNHG1 transcript or its genomic DNA sequence, both expressing dTomato. (**B**) Scheme of rescue of the depletion phenotype of SNHG1 KD cells. A673 cells were lipofected with transposon vectors containing either SNHG1 transcript or genomic DNA of SNHG1 gene. Then, cells were transduced with lentiviral sgNT or sgSNHG1 GFP^+^, which resulted in a mixed population of uninfected, GFP^+^, dTomato^+^, or GFP^+^/dTomato^+^ cells. Growth effect of GFP^+^/dTomato+ cell population was tracked by flow cytometry assessing ratios of these populations with the uninfected cells working as a normalizing internal control. (**C**) RT-qPCR analysis of SNHG1 expression in A673 dCas9-KRAB^+^ cells stably expressing the indicated SNHG1 construct and sgRNAs. Cells were harvested after sorting the GFP^+^/dTomato^+^ population. (**D**) Internally controlled growth assays performed in A673 cells stably expressing indicated SNHG1 construct. The GFP^+^/dTomato+ cell population was monitored over time by flow cytometry, with the uninfected cells working as a normalizing internal control (*n* = 3). Error bars in proliferation assays represent mean ± SEM. Relative transcripts levels in RT-qPCRs were normalized to HPRT1 then fold change sgRNA/sgNT was calculated. Error bars represent mean ± SD.

We restored the slow proliferation phenotype of SNHG1 KD by overexpressing this gene (Fig. 7A). A673 cells overexpressing the longest SNHG1 transcript (NR_003098.2, “cDNA SNHG1,” dTomato^+^), or the complete SNHG1 genomic sequence (“gDNA SNHG1,” lncRNA SNHG1 and intron-encoded snoRNAs genes, dTomato^+^) were transduced with lentivirus sgNT or sgSNHG1 (GFP^+^). The result was a heterogeneous population of single or double fluorescence positive cells (GFP^+^, dTomato^+^, or GFP^+^/dTomato^+^). We sorted the double positive population to determine the expression of SNHG1 by RT-qPCR and then we used the mixed population to perform an internally controlled growth assay tracking of double-positive cells by flow cytometry (Fig. 7B). We observed over-expression of SNHG1 transcripts in sgNT (four- to six-fold increase) and sgSNHG1 cells after transfection with either cDNA SNHG1 or gDNA SNHG1 (Fig. 7C). Notably, only over-expression of the SNHG1 gDNA completely rescued the proliferation phenotype of sgSNHG1 cells (Fig. 7D). In contrast the SNHG1 cDNA (lacking intron-encoded snoRNAs genes) did not rescue the low proliferation in EWS cells. This indicates that re-expression of the whole gene containing intron-encoded snoRNAs is required for rescue of the phenotype.

SNHG1 gene shares a promoter with the protein-coding gene *SLC3A2*, a cell surface transmembrane protein. As sgSNHG1 targets this bi-directional promoter, the observed proliferation phenotype in sgSNHG1 could be due to the decrease in the SLC3A2 expression. While we did observe a slight downregulation of SLC3A2 (Log_2_FC −0.33, adj. *P*-value .0341) and its associated Reactome pathway “Slc-mediated transmembrane transport” (NES −1.86 and adj. *P*-value 1.1 × 10^−3^) after 6 days after SNHG1 knockdown (Supplementary Fig. S11 and S14A), we did not observe any difference in SLC3A2 protein levels following the knockdown of SNHG1 (Supplementary Fig. S14B). We also reviewed the DepMap CRISPR screening dataset [43, 58] and found no dependencies for SLC3A2 knockout in EWS cells (Gene Effect-Chronos > −1.0, Supplementary Fig. S14C). This, together with our findings that overexpression of SNHG1 fully rescued the proliferation phenotype of SNHG1 KD, strongly suggests that the observed effect of knockdown of this gene is not due to positional effects on downregulation of SLC3A2. Similar experiments were attempted for SNHG12 and SNHG30, but overexpression of the corresponding cDNA was unsuccessful, therefore the rescue experiments were not performed.

### Alteration of snoRNA-guided posttranscriptional nucleoside modifications of ribosomal RNAs in SNHG1 and SNHG12 KD cells

SnoRNA guides play a critical role in introducing post-transcriptional modifications in RNAs. For example, the canonical function of the seven intronic snoRNAs (SNORD25-31) contained in SNHG1 is to guide 2′-O-methylation (Nm) modifications at specific sites in 28S and 18S rRNAs (Table 1) [59, 60], whereas the eighth snoRNA (SNORD22) directs processing of the 45S pre-rRNA [61]. Similarly, SNORD99 contained in SNHG12 gene directs Nm formation in 28S rRNA, while three other snoRNAs encoded in SNHG12 (SNORA61, SNORA44, and SNORA16A) guide rRNA pseudouridylation (Ψ) (Table 1) [60]. In contrast, SNORD7 from SNHG30 directs Nm modification of U6 snRNA [62–64], a core component of the spliceosome (Reviewed in Didychuk *et al.* [65]). Notably, the intronic snoRNAs are much more highly evolutionarily conserved regions than the exons of lncRNAs SNHG1, SNHG12, and SNHG30 (Supplementary Fig. S15A–C).

**Table 1.** Canonical functions of the snoRNAs encoded in SNHG1, 12, and 30 [66, 67]

snoRNA	RNA target	Type of chemical modification	Residue type and position
**SNHG1**			
SNORD22	necessary for efficient pre-rRNA processing		
SNORD31	rRNA 28S	Nm	Gm4166
SNORD30	rRNA 28S and 18S	Nm	Am3804 and Am1383
SNORD29	rRNA 28S	Nm	Am4493
SNORD28	rRNA 18S	Nm	Cm1391
SNORD27	rRNA 18S	Nm	Am27
SNORD26	rRNA 28S	Nm	Am389
SNORD25	rRNA 18S	Nm	Gm1490
**SNHG12**			
SNORD99	rRNA 28S	Nm	Am2774
SNORA61	rRNA 28S	Ψ	Ψ2495
SNORA44	rRNA 18S	Ψ	Ψ686 and Ψ822
SNORA16A	rRNA 28S	Ψ	Ψ4412
**SNHG30**			
SNORD7	snRNA U6	Nm	Am47

**Figure 8.**
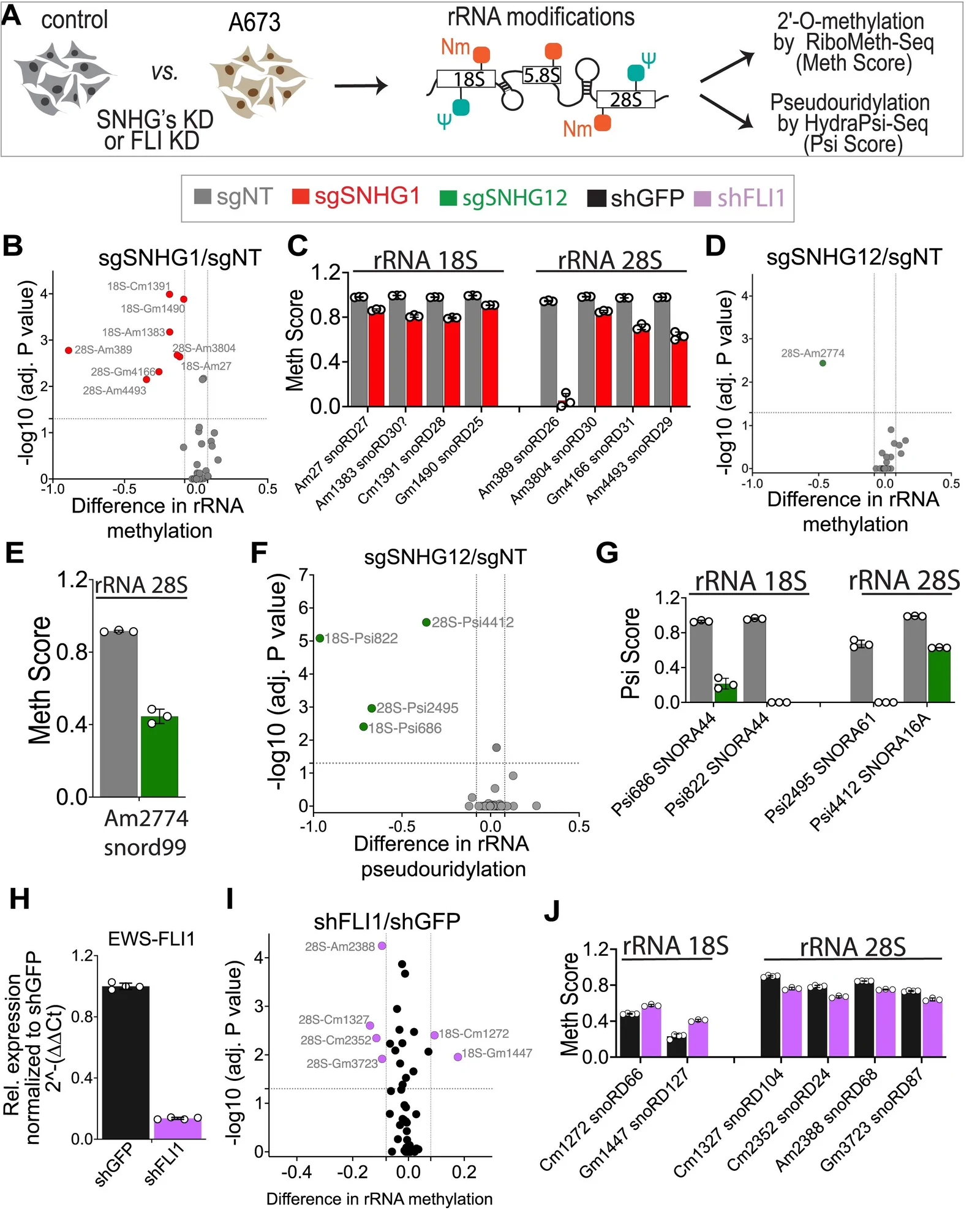
Post-transcriptional modifications in rRNAs of SNHG KD and shFLI1 KD cells. (**A**) Schema for the RiboMethSeq and HydraPsiSeq profiling. (**B**) rRNA 2′-O-methylation by RiboMethSeq comparing sgNT controls with sgSNHG1. (**C**) MethScores for the rRNA positions with statistically significant differences of MethScore between SNHG1 KD and controls. MethScore is a value from 0 to 1 reflecting the fraction of rRNA molecules 2′-O-methylated at a given position, where 1.0 indicates complete methylation [38]. (**D**) rRNA 2′-O-methylation by RiboMethSeq comparing sgNT controls with sgSNHG12. (**E**) MethScores values for the rRNA positions with significant difference of MethScore between SNHG12 KD and controls. (**F**) rRNA pseudouridylation by HydraPsiSeq comparing sgNT controls with sgSNHG12. (**G**) PsiScore values for the rRNA positions with significant difference of PsiScore between SNHG12 KD and controls. PsiScore reflects the fraction of rRNA pseudouridylated at a given position [41]. (**H**) RT-qPCR analysis of 3′ UTR FLI1 to quantify EWS–FLI1 oncogene transcript in A673 cells transduced with indicated shRNAs. Relative transcript levels in RT-qPCRs were normalized to HPRT1 then fold change shFLI1/shGFP was calculated. Error bars represent mean ± SD. (*n* = 3 or 4). (**I**) rRNA 2′-O-methylation by RiboMethSeq comparing shGFP controls with shFLI1. (**J**) MethScores for the rRNA positions with statistically significant difference of MethScore between FLI1 KD and controls. Error bars represent mean ± SD for each position. Statistically significant differences in rRNA methylation or pseudouridylation are defined as absolute of difference >0.08 and adj. *P*-value <.05. Adjusted *P*-values are from multiple unpaired *t*-tests, followed by Holm–Sidak’s multiple comparisons test. *n* = 3 or n = 4.

To evaluate the role of these snoRNAs in the SNHG-mediated regulation of EWS cell proliferation, we performed RiboMethSeq and HydraPsiSeq to quantify 2′-O-methylation (Nm) and pseudouridylation (Ψ) of rRNAs after SNHG KD in EWS cells (Fig. 8A) [38, 39]. Eight positions in the 18S and 28S rRNAs were detected as significantly hypomethylated in the SNHG1 KD cells, with the most affected being the 28S-Am389, which is known to be modified by SNORD26 (Fig. 8B and C, Supplementary Fig. S16A, and Supplementary Table S9) [59, 60, 66]. One location was hypomethylated (28S-Am2774/SNORD99) in the SNHG12 KD cells after knockdown (Fig. 8D and E, Supplementary Fig. S16B, and Supplementary Table S9). In contrast, there was no difference in the 2′-O-methylation profile of rRNA for SNHG30 KD compared with sgNT control (Supplementary Figs S16C and S18A, and Supplementary Table S9).

We also profiled the pseudouridylation level of previously reported positions in 18S and 28S rRNAs (Supplementary Fig. S17). Two positions in the 18S (Psi686 and Psi822, both guided by SNORA44) and other two in the 28S rRNA (Psi2495 guided by SNORA61 and Psi4412 guided by SNORA16A) were significantly hypo-pseudouridylated in the SNHG12 KD cells (Fig. 8F and G, Supplementary Fig. S17B, and Supplementary Table S10). There was no difference in the HydraPsiSeq profile for SNHG1 and SNHG30 KD compared with sgNT control (Supplementary Figs S17A and C, and S18B, and Supplementary Table S10). These results confirm that repression of SNHG1, SNHG12, and SNHG30 genes lead to decrease of specific chemical modifications in rRNAs. When the SNHG lncRNAs are knocked down, their snoRNAs housed within the introns are also downregulated, leading to a direct decrease in rRNA methylation at the sites.

To identify whether EWS–FLI1 influences the post-transcriptional modifications of rRNAs, we performed RiboMethSeq to quantify 2′-O-methylation (Nm) of rRNAs after EWS–FLI1 KD in Ewing cells (Fig. 8H). Two positions in the 18S rRNA were significantly hypermethylated (Cm1272 and Gm1447) upon EWS–FLI1 knockdown, while four positions in the 28S rRNA were significantly hypomethylated (Cm1327, Cm2352, Am2388, and Gm3723) (Fig. 8I and J, Supplementary Fig. S19, and Supplementary Table S11). Table 2 shows the snoRNAs that are predicted to guide these sites [59, 66, 67]. The rRNA Nm sites affected in EWS–FLI1 KD cells are distinct from those impacted in SNHG1, SNHG12, and SNHG30 KD cells.

**Table 2.** The 2′-O-methylation modifications regulated by EWS–FLI1 [66, 67]

Residue type and position	RNA target	snoRNA predicted to guide the modification	Host gene symbol
Cm1272	18S	SNORD66	EIF4G1
Gm1447	18S	SNORD127	PRPF39
Cm1327	28S	SNORD104	SNHG25
Cm2352	28S	SNORD24	RPL7A
Gm3723	28S	SNORD87	SNHG6
Am2388	28S	SNORD68	RPL13

## Discussion

The EWS–FLI1 oncoprotein profoundly alters the transcriptome of cells. Among the differentially expressed genes in EWS cells are a large number of lncRNAs. Indeed, as we demonstrate here, lncRNA expression is itself sufficient to cluster EWS compared to other pediatric cancers. While the role of EWS–FLI1-regulated pcg has been extensively studied, less is known regarding of the role of lncRNAs in the biology of EWS. To address this challenge, we first identified lncRNAs that are highly and specifically expressed in EWS compared with other pediatric cancers. A subset of these lncRNAs are regulated by EWS–FLI1, including EWSAT1, one of the most significantly differentially regulated lncRNAs by knockdown of EWS–FLI1 [14]. We comprehensively evaluated whether lncRNAs regulated by EWS–FLI1 or expressed in EWS have critical roles in cell fitness by carrying out a dropout CRISPRi screen. The vast majority of individual lncRNAs evaluated had no effect on proliferation of EWS cells. This was true also for EWSAT1 despite our prior studies indicating an important role for EWSAT1 in EWS–FLI1 driven oncogenesis. The reason for these divergent results is unclear, given that we have previously shown that shRNA-mediated knockdown of EWSAT1 has a significant effect on EWS–FLI1 regulated genes. Nevertheless, we found 35 lncRNA loci that, when transcriptionally repressed by dCas9-KRAB, lead to a decrease of cell fitness of Ewing cells. To our knowledge, the studies presented here are the most comprehensive evaluation of lncRNA function in EWS performed to date.

Several SNHG genes have been suggested to have a role in cancer [5, 68, 69]. We show that SNHG1, SNHG12, and SNHG30 impact EWS cell fitness, *in vitro* and *in vivo*. This vulnerability is likely due to the loss of the snoRNAs housed within the introns of the SNHG genes. GSEA analysis revealed enrichment of several paths related with the function of the snoRNAs encoded in the three SNHG genes (Table 1) [21]; for example, enrichment of “Ribosome Biogenesis,” “rRNA Assembly and Processing” paths in SNHG1 and SNHG12 KDs, and “Splicing” and “SNRPs complexes” in SNHG30 KD. SNHG30 encodes the snoRNA SNORD7, predicted to guide 2′-O-methylation of a residue (residue A47 in mice) in snRNA U6, a crucial component of spliceosomes [62, 64].

We demonstrated that knockdown of SNHG1 and SNHG12 decrease 2′-O-methylations and pseudouridylations at specific rRNA positions known to be guided by their snoRNAs (Table 1). For example, hypomethylation of Am2774 site in 28S rRNA guided by SNORD99, encoded in SNHG12, was observed in SNHG12 KD [59]. Overall, our findings indicate that the intron-processed snoRNAs genes rather than long SNHG transcripts might contribute to the maintenance of EWS cell fitness. Our transcriptome and post-transcriptional modifications data reveal a remarkable unbalance/dysregulation of rRNA biogenesis and processing that might lead to reduction of rRNA stability and function and/or to emergence of hypomethylated ribosomes having specific properties in mRNA translation. This therefore may be the underlying cause of the reduced fitness phenotype observed in the SNHG KD. Moreover, our work is the first study linking the SNHG KD phenotype in cell fitness with the role of their intron-encoded snoRNAs in rRNAs modifications.

We observed a cleaved PARP increase in A673 upon SNHG KD, which was not consistently observed in other EWS lines (Supplementary Fig. S10), arguing against acute apoptosis as the primary mechanism. We propose that the fitness defect is driven predominantly by a proliferative disadvantage arising from impaired ribosome biogenesis following snoRNA loss. Alternative mechanisms including autophagy or senescence may also contribute, both of which are known consequences of ribosomal stress [70, 71].

Within the SNHG family, one of the most studied members is SNHG1. SNHG1 is highly expressed in EWS compared with other pediatric cancers. EWS cells (A673, TC-32, and EW8) transduced with two different SNHG1 sgRNAs individually were rapidly depleted compared to uninfected cells confirming its role in cell fitness. SNHG1 also has a role in proliferation in osteosarcoma consistent with previous studies [72–75]. Prior studies have proposed that the mechanism of action of SNHG1 is through a role as a competitive “sponge” for microRNAs (reviewed in Huang *et al.* [76]). Another model is that SNHG1 functions as a transcriptional regulator acting both in *cis* and in trans. In *cis*, SNHG1 promotes transcription of SLC3A2, and in *trans*, it prevents FIR–FUBP1 protein interactions resulting in tuning of MYC expression levels in cancer cells [77]. SLC3A2 is a transmembrane protein that regulates calcium and l-type amino acid transport [78, 79]. Its TSS is 224 bp from SNHG1, so dCas9-KRAB repression could affect SLC3A2 expression [16, 29, 30]. This is reflected in GSEA, where SLC-mediated transmembrane transporter activity is reduced in SNHG1 KD (NES −1.86 and adj *P-value* = 1.1 × 10^−3^) (Supplementary Fig. S11). Despite this, ectopic SNHG1 overexpression fully rescued the phenotype, showing SNHG1 is key to proliferation. However, we cannot fully disentangle the contributions of SNHG1 and SLC3A2, since SNHG1 has been shown to regulate SLC3A2 in other cancer types [77] and ectopic SNHG1 re-expression may co-restore SLC3A2 levels.

Rescue experiments for SNHG12 and SNHG30 were not feasible in this study. Nevertheless, all three SNHG KDs produce convergent depletion of ribosome biogenesis and rRNA processing gene sets by GSEA, supporting a shared snoRNA-dependent mechanism. For SNHG12, this is further reinforced by direct functional evidence: SNORD99 co-downregulation (Fig. 6D), an intronic snoRNA encoded in the last intron of SNHG12, notably the most distal snoRNA from the CRISPRi sgRNA target sites, indicating that transcriptional repression propagates through the entire locus. Loss of rRNA modifications was observed at all four snoRNA-guided positions upon KD (Fig. 8D–G). However, genomic rescue constructs for SNHG12 and SNHG30 with or without mutations at the snoRNA loci remain an important future direction to formally demonstrate that the snoRNAs are required for the proliferation phenotype.

The RNA-seq data for SNHG12 knockdown showed enrichment of several pathways involving cardiovascular and circulatory system. These results are consistent with previous findings showing that SNHG12 is highly expressed in the vascular endothelium, it regulates the DNA damage response and senescence in the vascular endothelium [80]. Moreover, SNHG12 regulates genes related with DNA damage responses including FIGNL1 [81] and TMEM161A [82]. Further studies will need to be done to evaluate the specific role of SNHG12 in regulating the DNA damage response in EWS.

Six Nm rRNA positions were altered in EWS–FLI1 KD. These methylation sites have been reported to belong to the group of highly variable sites where the level of their methylation shows high variance across a panel of 15 human cell lines [40]. This group of variable Nm sites is linked to the concept of “specialized ribosomes,” which can modulate translation of certain mRNAs and affect interactions with tRNAs or regulatory factors associated with ribosomes [83]. The difference of the levels of 2′-O-methylation observed for these rRNA sites could be result of alterations in the expression of components of the 2′-O-methylation complex such as specific snoRNA guides or the rRNA methyl-transferase FBL [84]. Published data [85] previously identified FBL as part of the consensus EWS signature. Therefore, EWS–FLI1 might influence rRNA methylation by direct regulation of FBL expression, or indirectly by modulating other factors involved in rRNA processing or modification. In summary, our study suggests that expression of SNHG1, SNHG12, and SNHG30 in EWS cells contributes to the maintenance of cancer cell fitness via their intron-processed snoRNA genes. Thus, post-transcriptional control of rRNA functions could be an overlooked and novel function of EWS–FLI1.

## Supplementary Material

zcag019_Supplemental_Files

## Data Availability

The CRISPRi screen and RNA sequencing data underlying this article are available in NCBI GEO (https://www.ncbi.nlm.nih.gov/geo/) and can be accessed with accession number GSE267996.
