# Supplementary material for: SNHG family lncRNAs promote Ewing sarcoma cell fitness through intronic snoRNA-guided rRNA modifications
Source: NAR Cancer. 2026 Aug 26;8(3):zcag019. doi: 10.1093/narcan/zcag019 (PMC13507652; doi:10.1093/narcan/zcag019)
Supplement: Supplementary file 1 [file zcag019_supplemental_files.zip › SupplementaryFiguresMB8_REVISED.pdf]

Supplementary Figures for:

SNHG family lncRNAs promote Ewing sarcoma cell fitness through intronic snoRNA-guided rRNA modifications

Marcela Briones-Martin-del-Campo[1], Alex G. Lee[1], Truc Dinh[1], Virginie Marchand[2], Yuri Motorin[3], Marta Román[1], Qiuli Bi[1], Stanley G. Leung[1], Max A. Horlbeck[4], Leanne C. Sayles[1], Bo Kyung Alex Seong[5], Luke Gilbert[6], Kimberly Stegmaier[5], E. Alejandro Sweet-Cordero[1*]

[1] Department of Pediatrics, University of California, San Francisco, CA 94158, USA.

[2] Epitranscriptomics and RNA Sequencing Core Facility, Université de Lorraine, CNRS, INSERM, IBSLor (UAR2008/US40), Nancy F-54000, France.

[3] IMoPA (UMR7365), CNRS, Université de Lorraine, Nancy F-54000, France.

[4] Department of Pediatrics, Division of Genetics and Genomics, Boston Children's Hospital, Boston, MA 02115, USA.

[5] Department of Pediatric Oncology, Dana-Farber Cancer Institute and Boston Children’s Hospital, Boston, MA, USA.

[6] Department of Urology, University of California, San Francisco, CA 94158, USA.

* To whom correspondence should be addressed. Tel: +1 415-476-7781; Email: Alejandro.Sweet-Cordero@ucsf.edu

This file includes:

Supplementary Figures S1 to S19

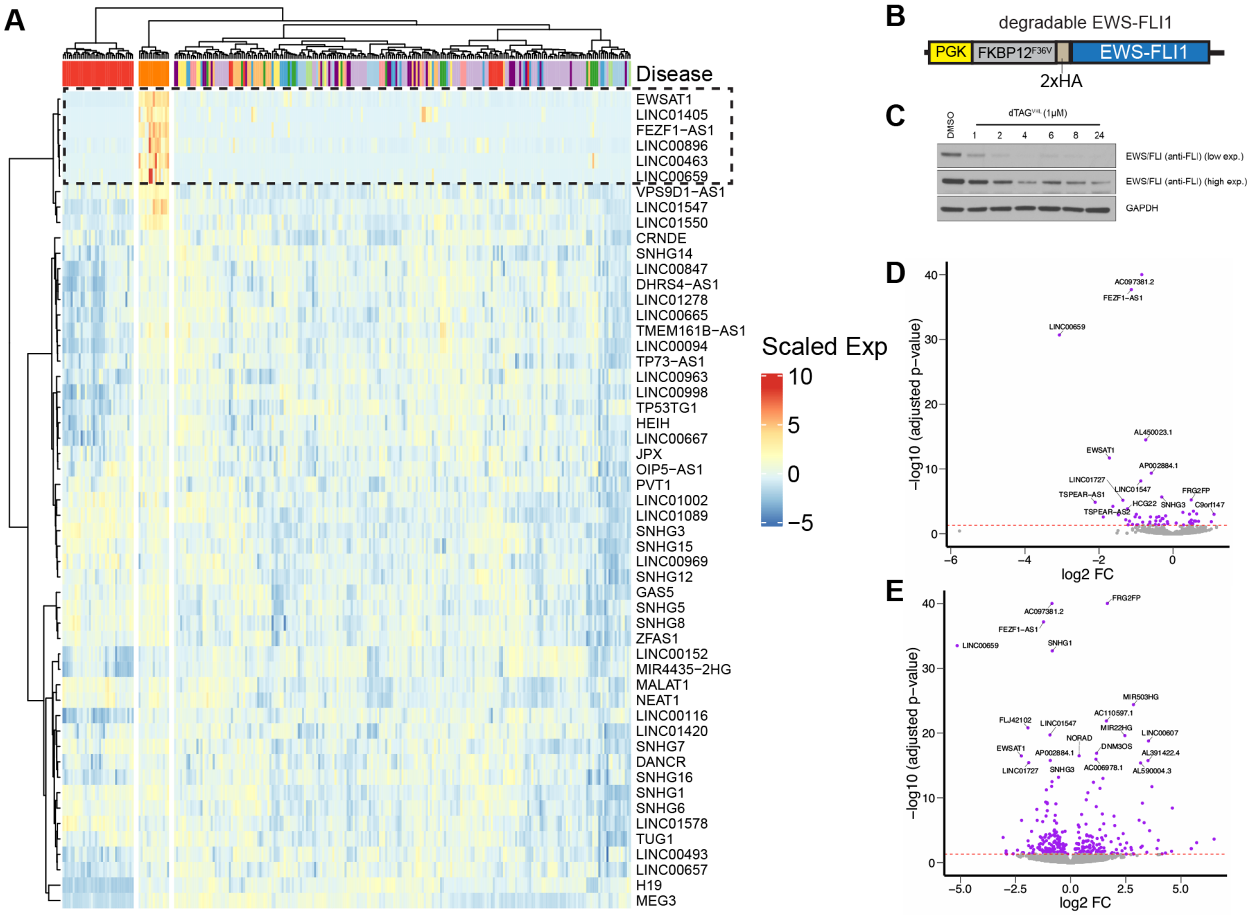

Supplementary Figure S1. (**A**) Heatmap displaying the top 50 lncRNAs with the highest expression in EWS. Colored bars at the top represent eleven different pediatric cancers, including 15 EWS in orange. The lncRNA genes within a dotted rectangle are the ones expressed relatively more highly in EWS than in other diseases. They are also shown in separate individual plots in Figure 1B. (**B**) Scheme of the degradable EWS–FLI1 construct. (**C**) Western blot of FKBP12^{F36V} tagged EWS–FLI1 in protein extracts of EWS502 EWS–FLI1$^{-/-}$ cells treated with either DMSO or 1µM of dTAG^{VHL} at different timepoints. GAPDH was used as a loading control. (**D**) Volcano plot of ncRNA genes upregulated and downregulated in EWS502 FKBP12^{F36V}-EWS–FLI1 cells treated with either DMSO or 1 µM of dTAG^{VHL} for 6 h. Purple dots represent ncRNA genes with FDR < 0.05. (**E**) Similar to (D) but dTAG^{VHL} for 72 h.

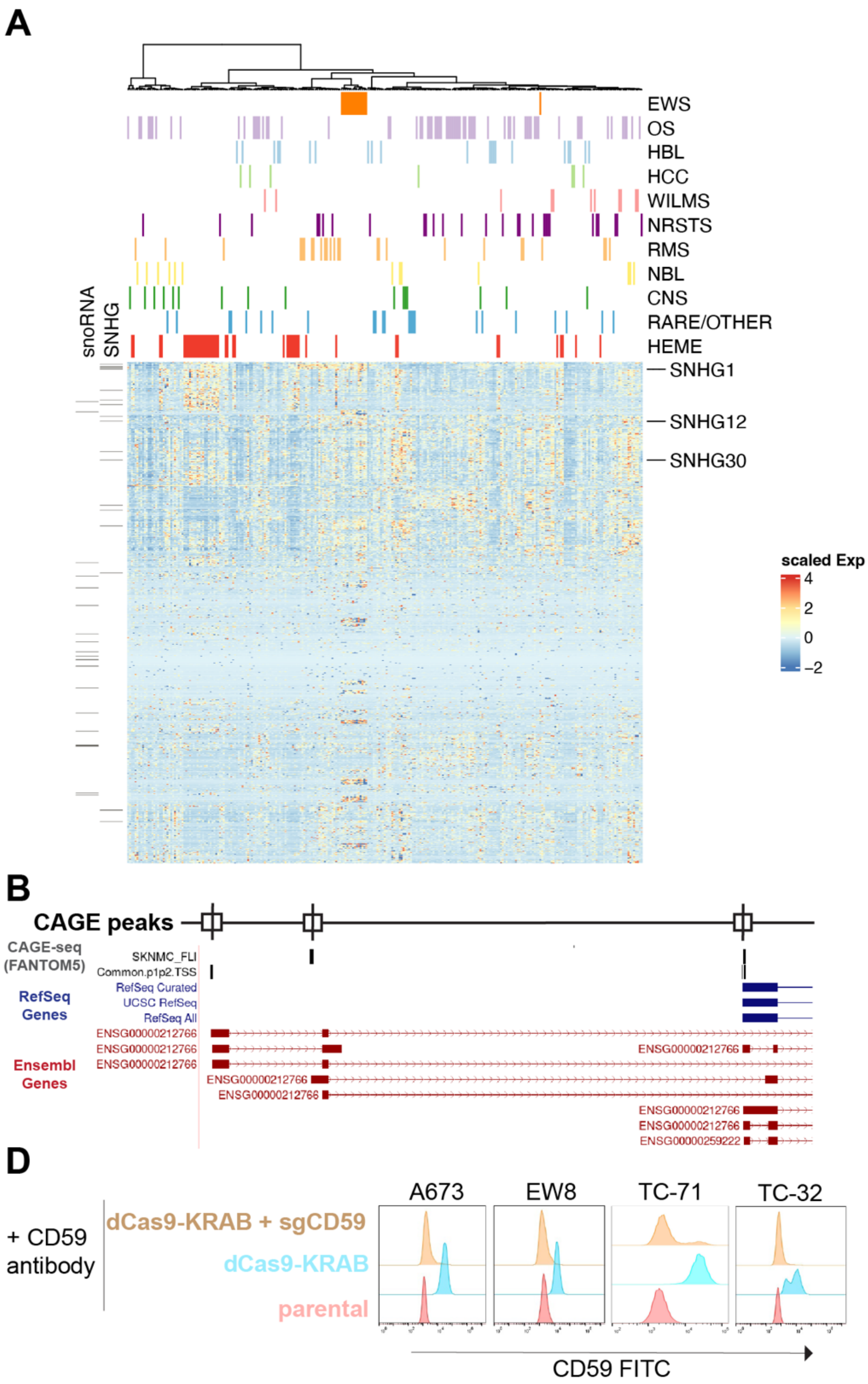

Supplementary Figure S2. (**A**) Cluster analysis and heatmap of genes included in the CRISPRi screen in a large collection of primary tumors. Colored bars at the top represent eleven different pediatric cancers, including 15 EWS in orange. (**B**) Example for Ewing-specific TSS prediction by FANTOM5 CAGE peaks analysis. (**C**) Distribution of TSSs per locus targeted by the sgRNA library. TSSs were defined based on CAGE peaks. (**D**) CRISPRI knockdown efficiency of Ewing Sarcoma cell lines was assayed using sgRNA against CD59 gene. Flow cytometry plots showing intensity of CD59 FITC antibody staining in dCas9-KRAB cells transduced with sgCD59 (beige), dCas9-KRAB cells (blue) and parental cell lines (red) as controls.

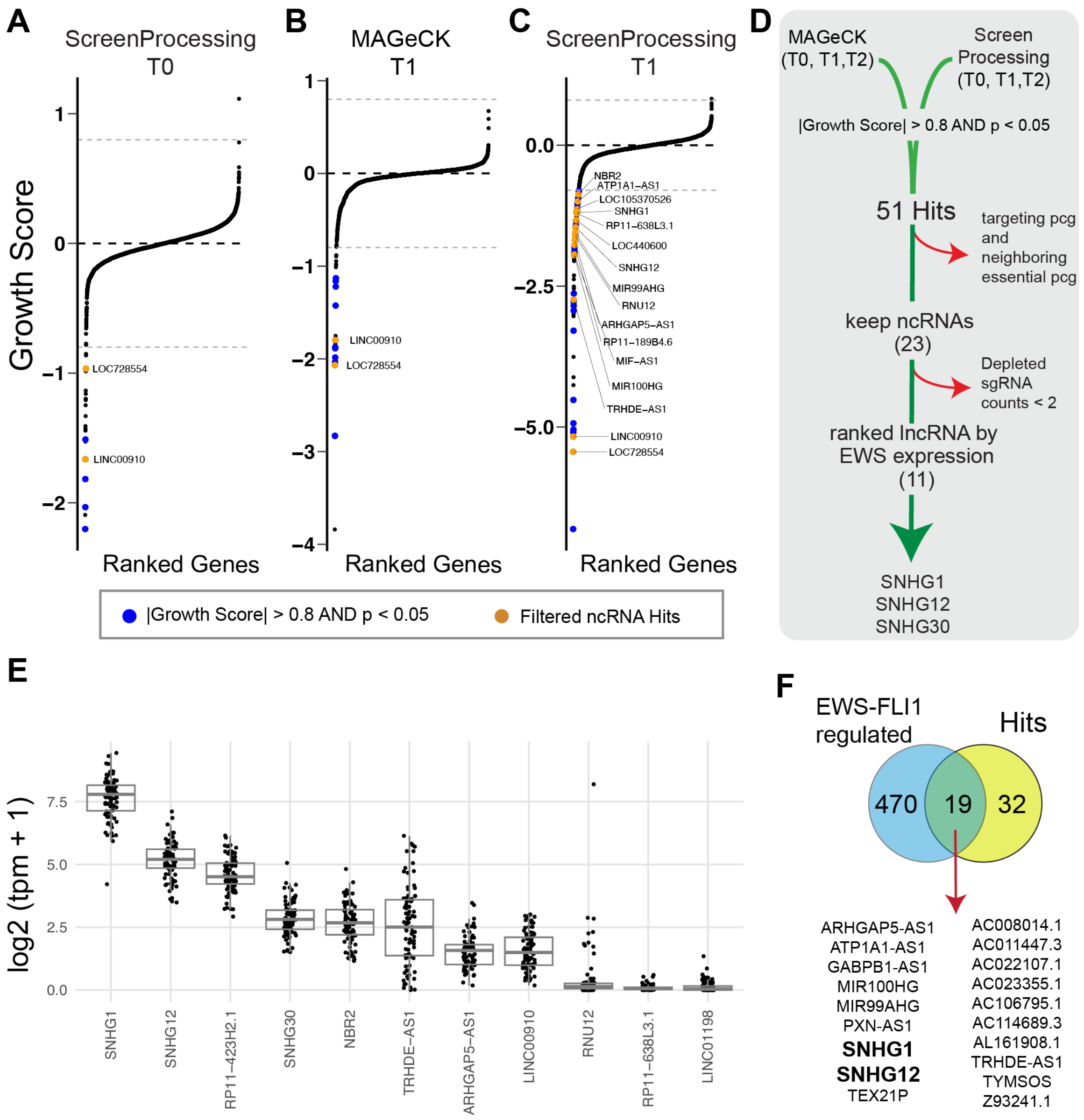

Supplementary Figure S3. (**A–C**) Growth screen results showing gene rankings based on their impact on proliferation of A673 dCas9-KRAB^{+} cells. (**A**) Results from timepoint 0 (T0) analyzed using ScreenProcessing. (**B**) Results from timepoint 1 (T1) analyzed using MAGeCK. (**C**) Results from timepoint 1 (T1) analyzed using ScreenProcessing. Genes with significant effects (|Growth Score| > 0.8 and p < 0.05) are shown in blue. Key filtered lncRNA hits are highlighted in orange (prioritized as described in panel D), and all other genes are shown in black. Statistical significance was determined using adjusted Wald-FDR p-values for MAGeCK and Mann–Whitney p-values for ScreenProcessing. (**D**) Filtering and prioritization strategy of hits. Out of 51 initial hits, 23 were prioritized (highlighted in orange in A–C) by excluding those targeting protein-coding genes (pcg) and those near essential pcg, retaining lncRNAs with at least two sgRNAs showing significant depletion, and selecting for those with EWS expression (shown in E). Three top candidate SNHG family lncRNAs were prioritized for validation. (**E**) Expression of prioritized lncRNA hits across EWS patient tumor samples from TCCI collection. (**F**) Venn diagram showing the intersection of CRISPR screen hits with lncRNAs differentially expressed upon EWS–FLI1 degradation (FDR < 0.05 at 6, 24, or 72 hours post-dTAG^{VHL} treatment) in EWS502 FKBP12^{F36V}-EWS–FLI1 cells. Nineteen ncRNAs were identified as both screen hits and EWS–FLI1-regulated.

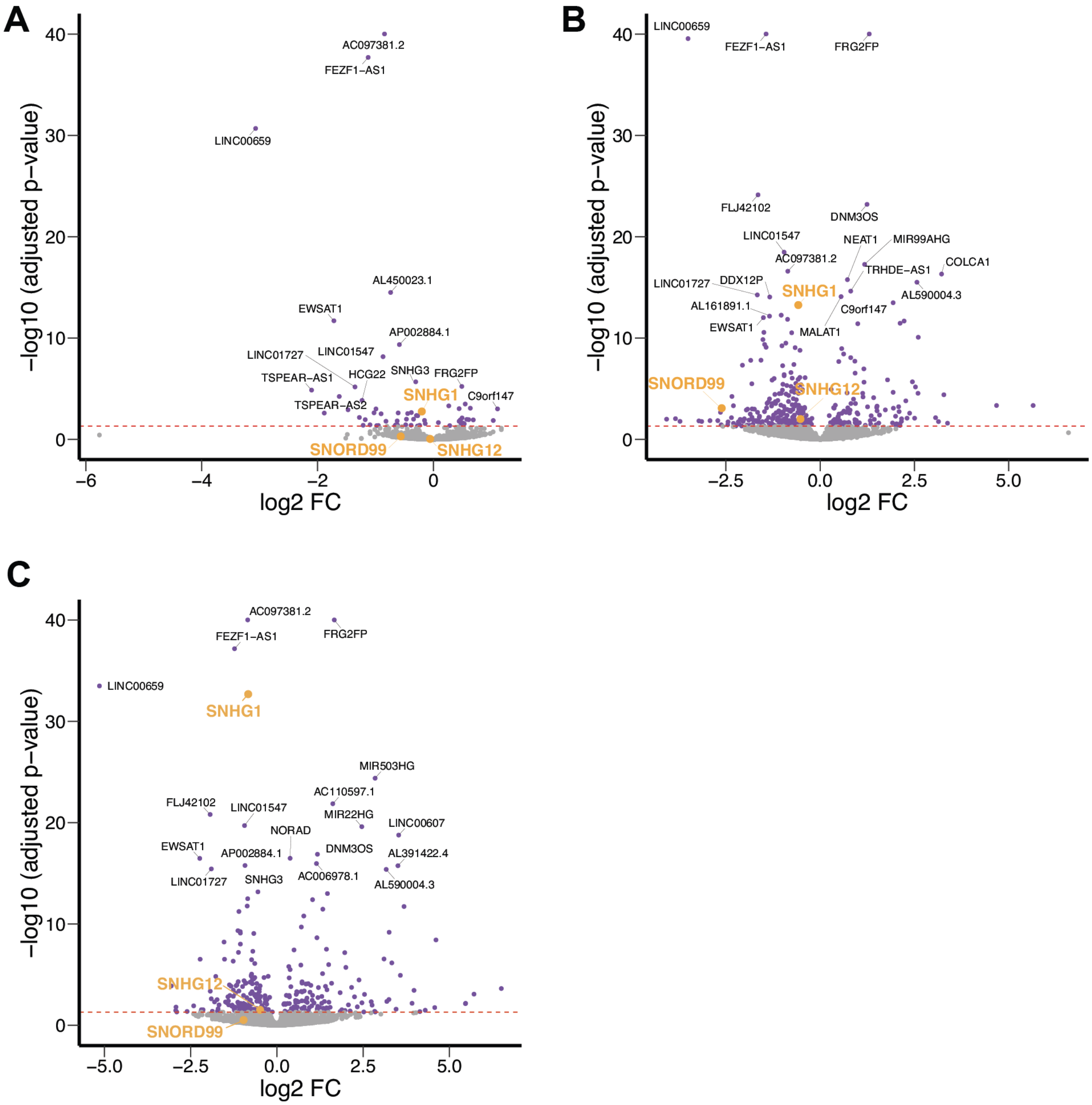

Supplementary Figure S4. (**A**) Volcano plot of ncRNA genes upregulated and downregulated in EWS502 FKBP12^{F36V}-EWS–FLI1 cells treated with either DMSO or 1 µM of dTAG^{VHL} for 6 h. Purple dots represent ncRNA genes with FDR < 0.05. SNHG1, SNHG12 and the SNHG12-intronic snoRNA SNORD99 are highlighted in orange. (**B**) Similar to (A) but dTAG^{VHL} for 24 h. (**C**) Similar to (A) but dTAG^{VHL} for 72 h.

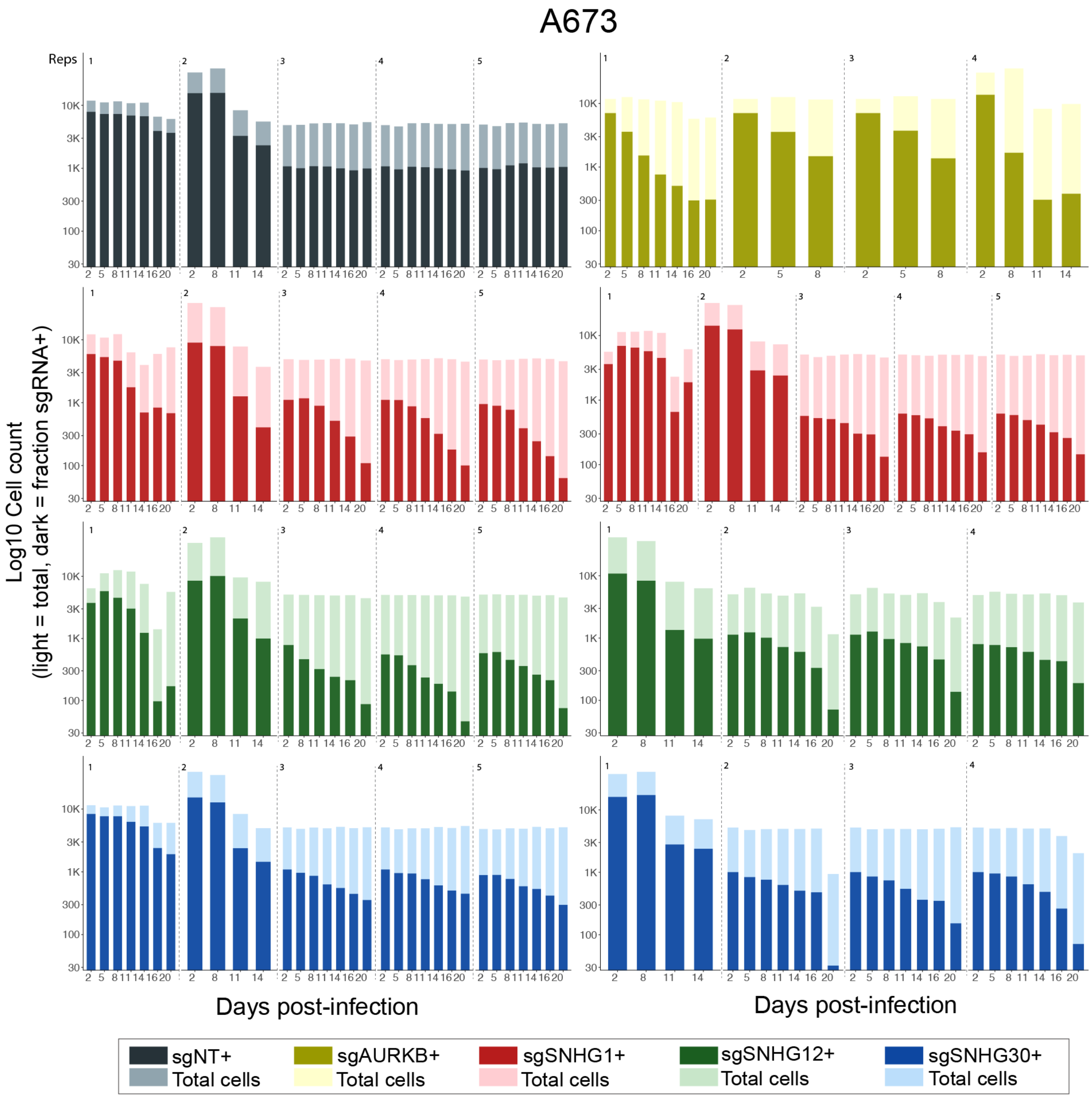

Supplementary Figure S5. Log10-scaled cell counts of sgRNA+ fraction (dark bars) and total cell population (light bars) over 20 days post-lentiviral infection in A673 cells, as quantified by flow cytometry. Data shown for up to five independent biological replicates per sgRNA.

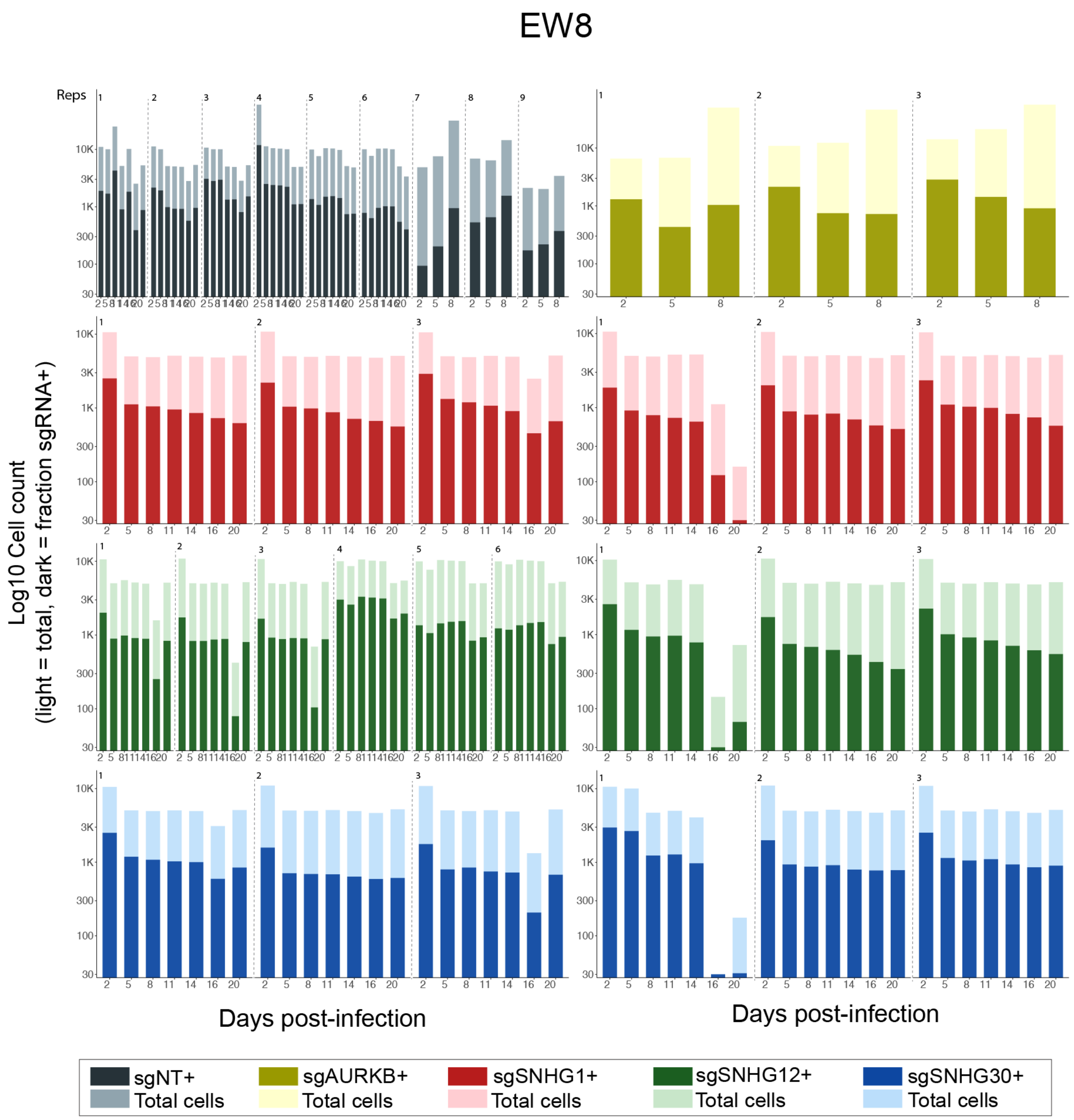

Supplementary Figure S6. Log10-scaled cell counts of sgRNA+ fraction (dark bars) and total cell population (light bars) over 20 days post-lentiviral infection in EW8 cells, as quantified by flow cytometry. Data shown for up to nine independent biological replicates per sgRNA.

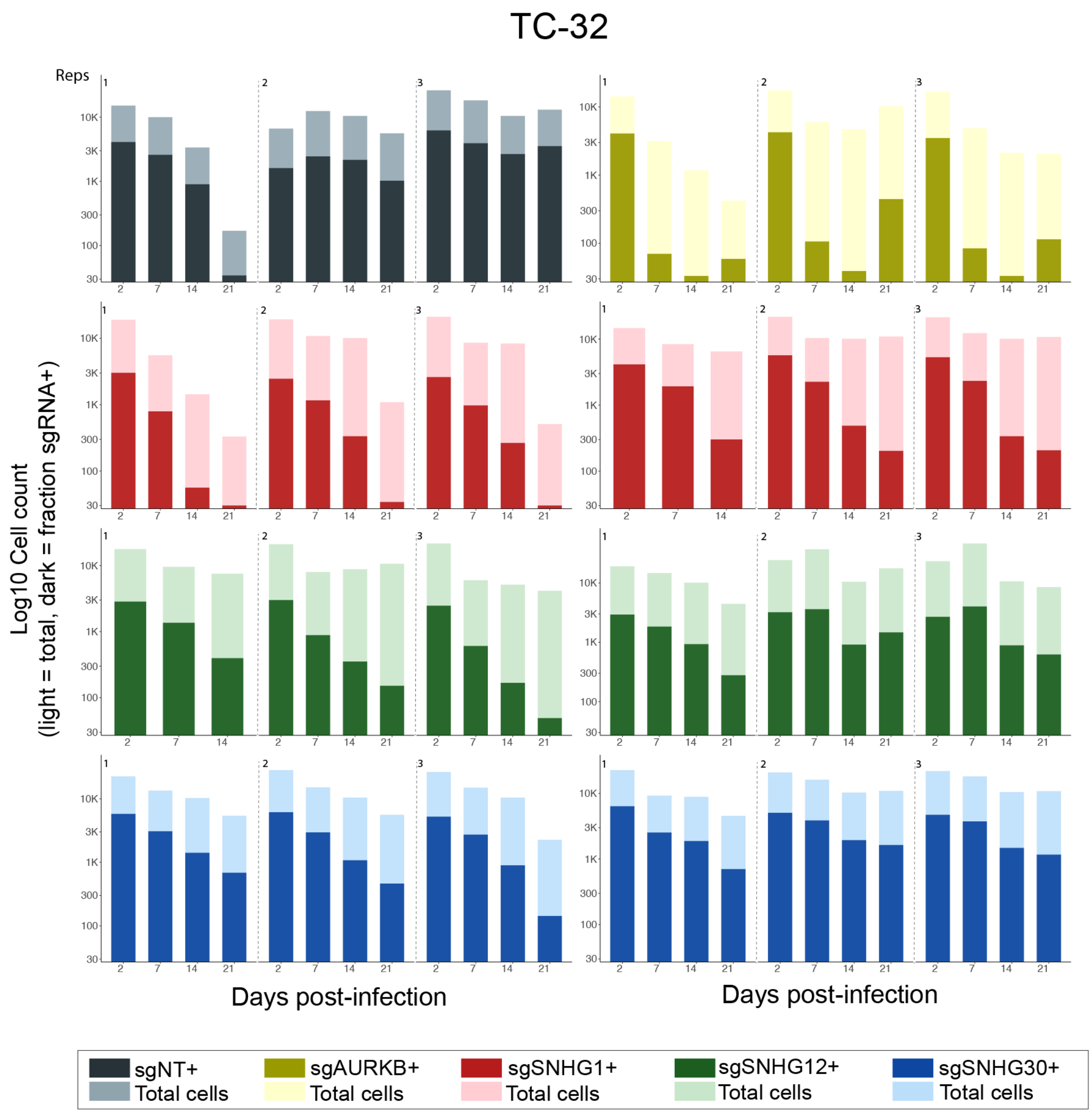

Supplementary Figure S7. Log10-scaled cell counts of sgRNA+ fraction (dark bars) and total cell population (light bars) over 20 days post-lentiviral infection in TC-32 cells, as quantified by flow cytometry. Data shown for up to three independent biological replicates per sgRNA.

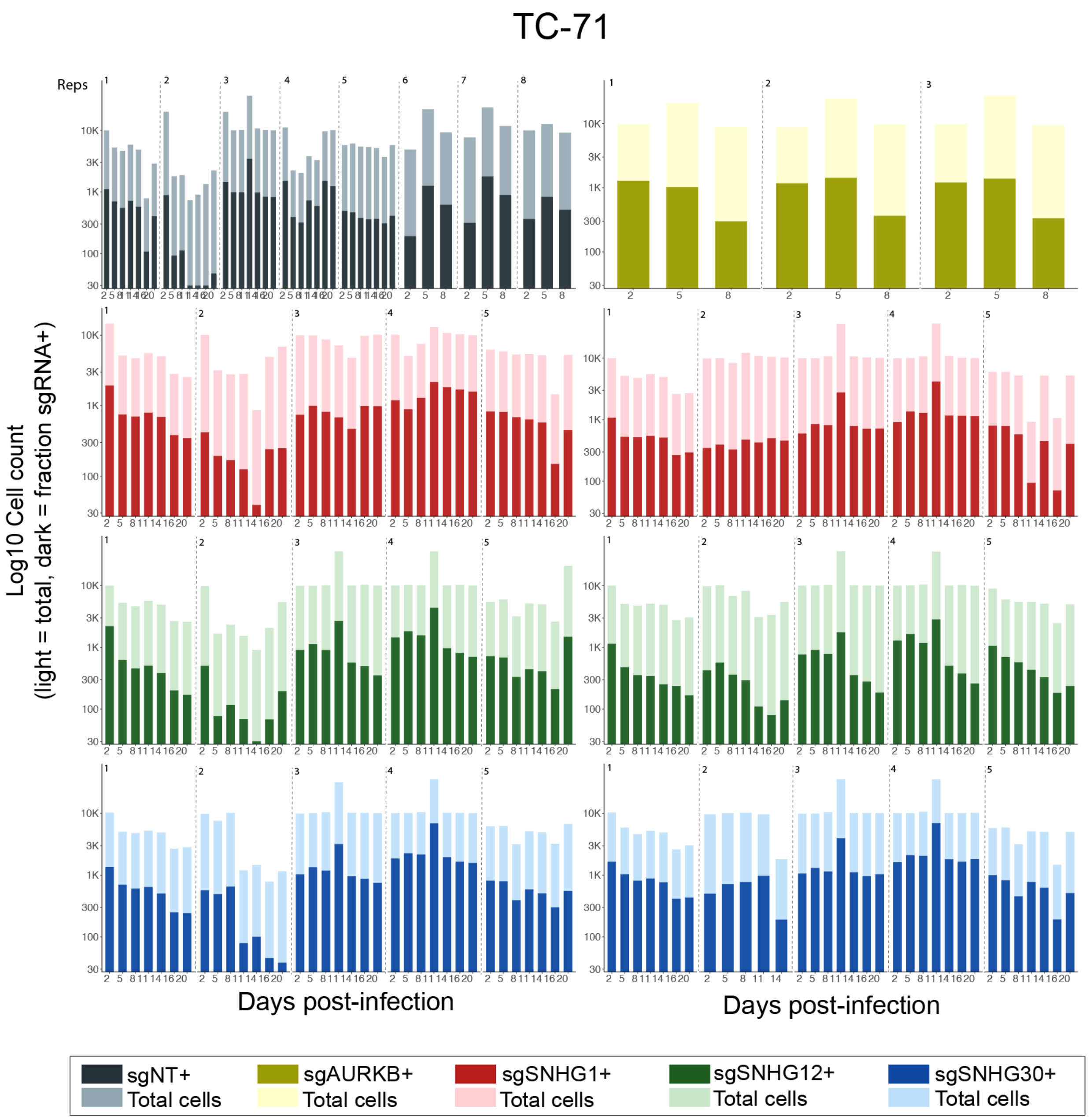

Supplementary Figure S8. Log10-scaled cell counts of sgRNA+ fraction (dark bars) and total cell population (light bars) over 20 days post-lentiviral infection in TC-71 cells, as quantified by flow cytometry. Data shown for up to eight independent biological replicates per sgRNA.

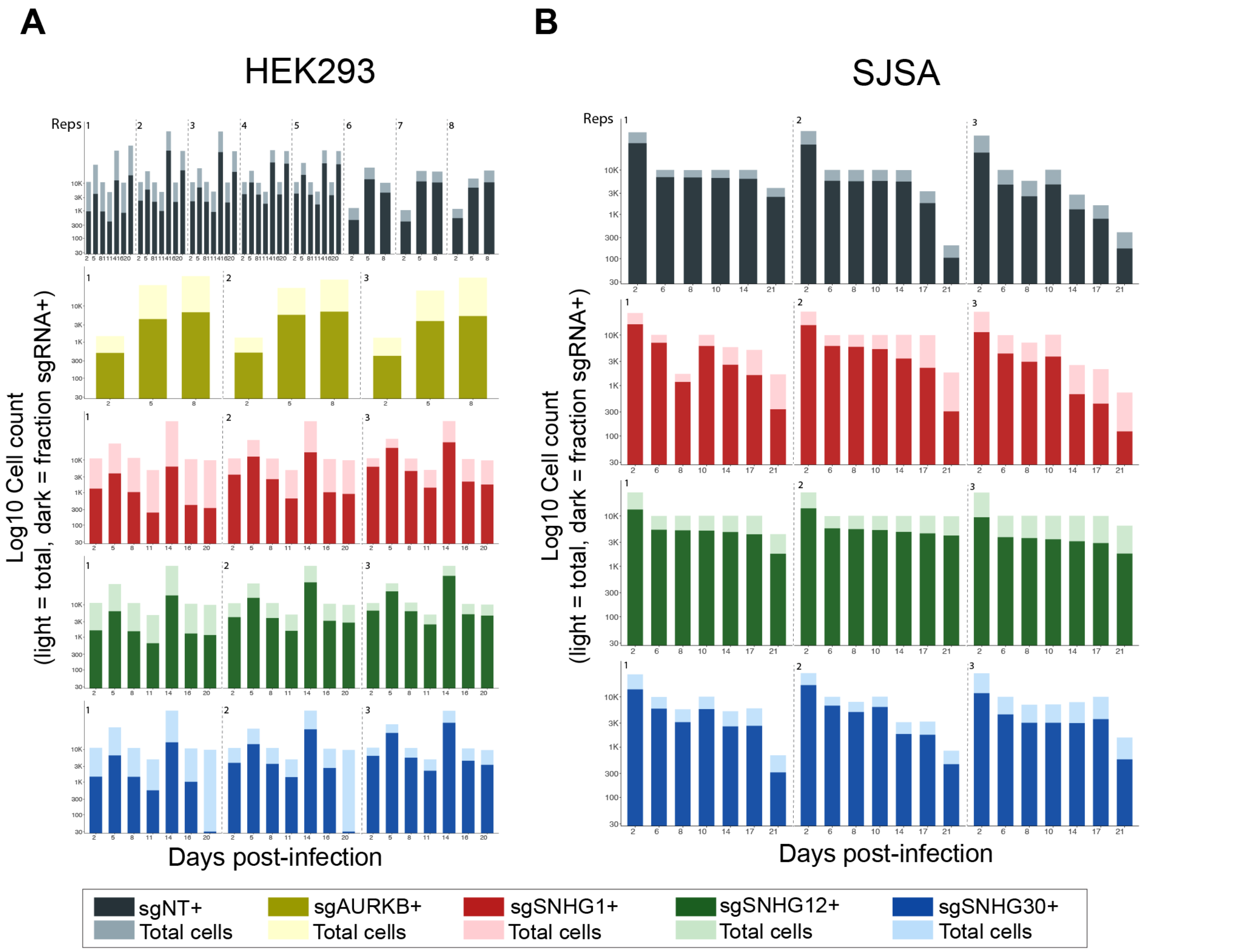

Supplementary Figure S9. Log10-scaled cell counts of sgRNA+ fraction (dark bars) and total cell population (light bars) over 20 days post-lentiviral infection in (**A**) HEK293 cells and (**B**) SJSA, as quantified by flow cytometry. Data shown for up to eight independent biological replicates per sgRNA for HEK293 and three for SJSA.

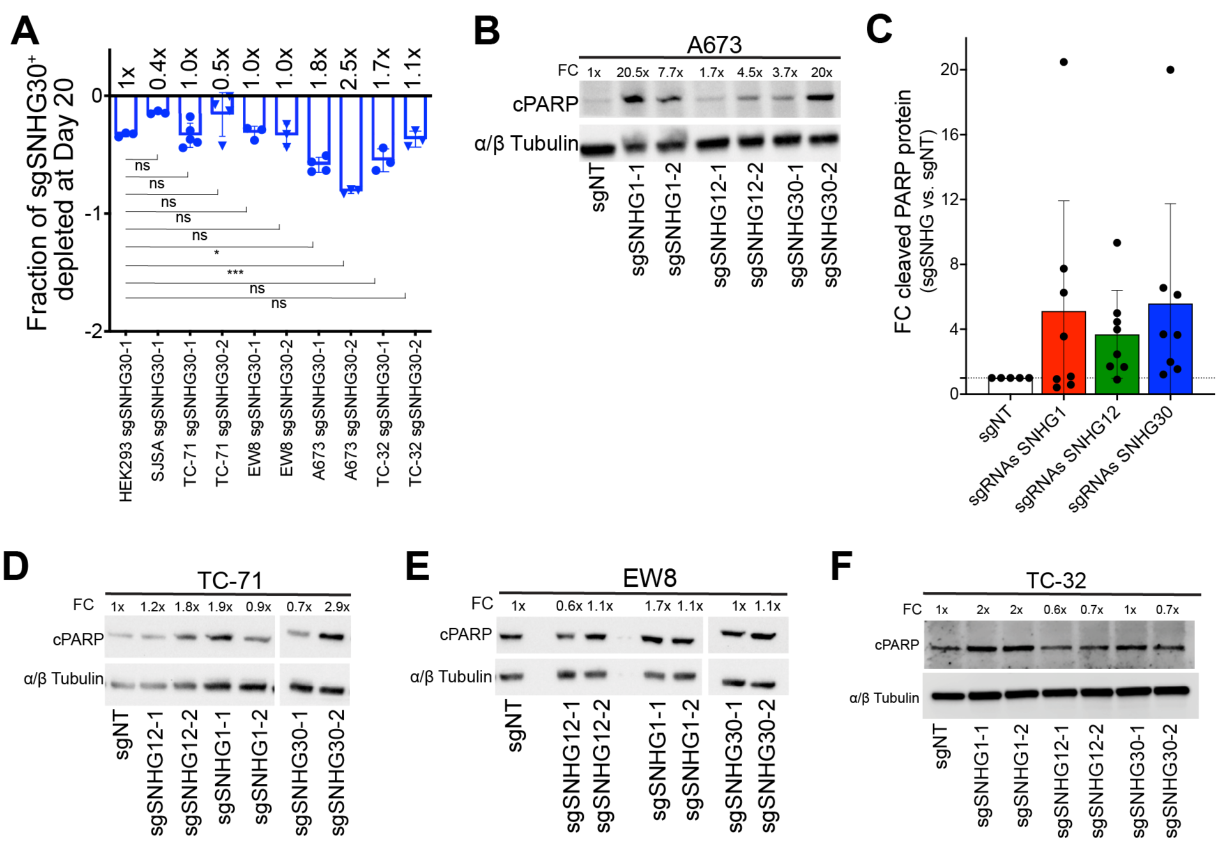

Supplementary Figure S10. (**A**) Negative values indicate the fraction of sgSNHG30$^+$ cells depleted at Day 20 for each dCas9-KRAB$^+$ cell line. Each bar plot is labeled with the specific fold change of sgSNHG30$^+$ depletion versus HEK293. Data represent the mean ± SD. One-way ANOVA followed by Dunnett's multiple comparison test where "ns" indicates nonsignificant (p-value > 0.05); * denotes p-value ≤ 0.05, ** denotes p-value ≤ 0.01, *** denotes p-value ≤ 0.001. (**B–F**) Western blot analysis of cleaved PARP (cPARP) protein levels for dCas9-KRAB$^+$ cell lines transduced with the indicated sgRNAs. α/β-Tubulin was used as a loading control. Numbers above each lane indicate fold change (FC) relative to sgNT, calculated after normalization of cPARP signal to α/β-Tubulin. Western blots are shown for (**B**) A673, (**D**) TC-71, (**E**) EW8, and (**F**) TC-32 cells. (**C**) Densitometry for A673 across westerns, bars show mean ± SD.

A673 dCas9-KRAB+ sgSNHG1 *vs.* sgNT

Pathway	Gene ranks	NES	pval	padj
REACTOME_SLC_MEDIATED_TRANS...		–1.86	$1.5 \cdot 10^{-5}$	$1.1 \cdot 10^{-3}$
BP_MRNA_METABOLIC_PROCESS		2.02	$1.0 \cdot 10^{-10}$	$4.0 \cdot 10^{-8}$
REACTOME_METABOLISM_OF_RNA		1.88	$1.0 \cdot 10^{-10}$	$4.0 \cdot 10^{-8}$
REACTOME_PROCESSING_OF_CAPP...		2.08	$3.2 \cdot 10^{-10}$	$1.0 \cdot 10^{-7}$
BP_RIBONUCLEOPROTEIN_COMPLE...		2.06	$1.0 \cdot 10^{-10}$	$4.0 \cdot 10^{-8}$
BP_NCRNA_PROCESSING		2.05	$1.0 \cdot 10^{-10}$	$4.0 \cdot 10^{-8}$
BP_RRNA_METABOLIC_PROCESS		2.02	$2.0 \cdot 10^{-8}$	$3.7 \cdot 10^{-6}$
REACTOME_RRNA_MODIFICATION_...		2.00	$9.5 \cdot 10^{-5}$	$4.2 \cdot 10^{-3}$
BP_CHROMOSOME_ORGANIZATION		1.54	$1.0 \cdot 10^{-10}$	$4.0 \cdot 10^{-8}$
CC_RIBONUCLEOPROTEIN_COMPLEX		1.69	$1.0 \cdot 10^{-10}$	$4.0 \cdot 10^{-8}$

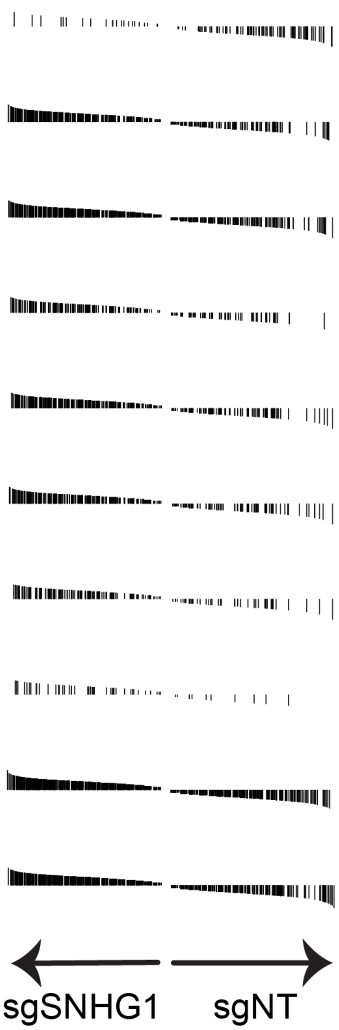

Supplementary Figure S11. Enriched pathways in SNHG1 KD cells. Table of enriched pathways for A673 SNHG1 KD identified by GSEA. The following gene sets were used: GO terms (BP, CC or MF), Reactome, KEGG, and Hallmark of Cancer.

A673 dCas9-KRAB+ sgSNHG12 *vs.* sgNT

Pathway	NES	pval	padj
REACTOME_NONSENSE_MEDIATED_...	−2.39	$7.2 \cdot 10^{-8}$	$5.9 \cdot 10^{-6}$
KEGG_RIBOSOME	−2.42	$1.2 \cdot 10^{-7}$	$9.0 \cdot 10^{-6}$
REACTOME_EUKARYOTIC_TRANSLA...	−2.28	$3.5 \cdot 10^{-7}$	$2.4 \cdot 10^{-5}$
BP_NUCLEAR_TRANSCRIBED_MRNA...	−2.21	$3.1 \cdot 10^{-7}$	$2.2 \cdot 10^{-5}$
REACTOME_NONSENSE_MEDIATED_...	−2.39	$7.2 \cdot 10^{-8}$	$5.9 \cdot 10^{-6}$
MF_STRUCTURAL_CONSTITUENT_O...	−2.16	$1.8 \cdot 10^{-6}$	$8.9 \cdot 10^{-5}$
REACTOME_RRNA_PROCESSING	−1.94	$5.8 \cdot 10^{-6}$	$2.3 \cdot 10^{-4}$
BP_POSITIVE_REGULATION_OF_V...	2.58	$1.0 \cdot 10^{-10}$	$1.6 \cdot 10^{-8}$
BP_REGULATION_OF_VASCULATUR...	2.38	$1.0 \cdot 10^{-10}$	$1.6 \cdot 10^{-8}$
BP_CIRCULATORY_SYSTEM_DEVEL...	1.94	$1.0 \cdot 10^{-10}$	$1.6 \cdot 10^{-8}$
BP_CARDIOVASCULAR_SYSTEM_DE...	1.93	$1.0 \cdot 10^{-10}$	$1.6 \cdot 10^{-8}$
HALLMARK_EPITHELIAL_MESENCH...	2.95	$1.0 \cdot 10^{-10}$	$1.6 \cdot 10^{-8}$
HALLMARK_UV_RESPONSE_DN	2.32	$7.5 \cdot 10^{-10}$	$10.0 \cdot 10^{-8}$
HALLMARK_APOPTOSIS	1.97	$2.1 \cdot 10^{-6}$	$1.0 \cdot 10^{-4}$
HALLMARK_HYPOXIA	1.98	$1.3 \cdot 10^{-6}$	$6.6 \cdot 10^{-5}$

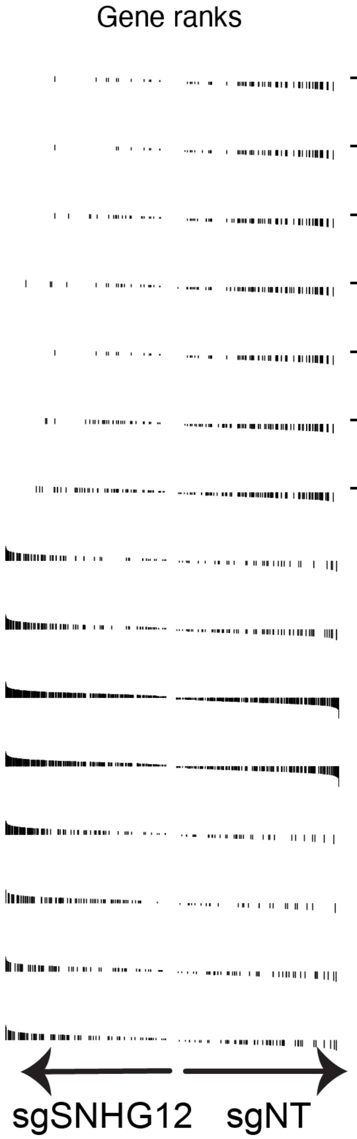

Supplementary Figure S12. Enriched pathways in SNHG12 KD cells. Table of enriched pathways for A673 SNHG12 KD identified by GSEA. The following gene sets were used: GO terms (BP, CC or MF), Reactome, KEGG, and Hallmark of Cancer.

A673 dCas9-KRAB+ sgSNHG30 *vs.* sgNT

Pathway	NES	pval	padj
REACTOME_MRNA_SPLICING	2.43	$1.0 \cdot 10^{-10}$	$3.5 \cdot 10^{-8}$
BP_RNA_SPLICING	2.20	$1.0 \cdot 10^{-10}$	$3.5 \cdot 10^{-8}$
BP_MRNA_METABOLIC_PROCESS	2.15	$1.0 \cdot 10^{-10}$	$3.5 \cdot 10^{-8}$
REACTOME_METABOLISM_OF_RNA	2.08	$1.0 \cdot 10^{-10}$	$3.5 \cdot 10^{-8}$
BP_RNA_METABOLIC_PROCESS	1.97	$1.0 \cdot 10^{-10}$	$3.5 \cdot 10^{-8}$
BP_NCRNA_PROCESSING	1.76	$2.6 \cdot 10^{-8}$	$5.1 \cdot 10^{-6}$
BP_NCRNA_METABOLIC_PROCESS	1.70	$6.9 \cdot 10^{-8}$	$1.1 \cdot 10^{-5}$
BP_RRNA_METABOLIC_PROCESS	2.00	$6.9 \cdot 10^{-8}$	$1.1 \cdot 10^{-5}$
REACTOME_RRNA_PROCESSING	1.64	$9.4 \cdot 10^{-5}$	$5.4 \cdot 10^{-3}$
CC_CATALYTIC_STEP_SPLICEOSOME	2.36	$3.6 \cdot 10^{-8}$	$6.5 \cdot 10^{-6}$

Gene ranks

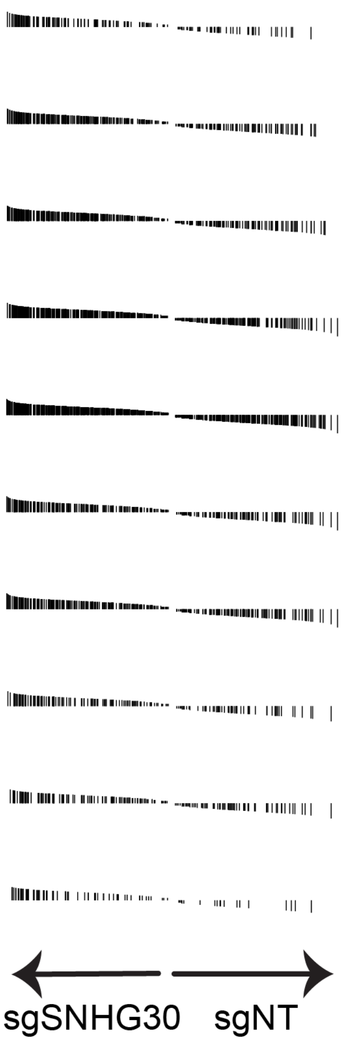

Supplementary Figure S13. Enriched pathways in SNHG30 KD cells. Table of enriched pathways for A673 SNHG30 KD identified by GSEA. The following gene sets were used: GO terms (BP, CC or MF), Reactome, KEGG, and Hallmark of Cancer.

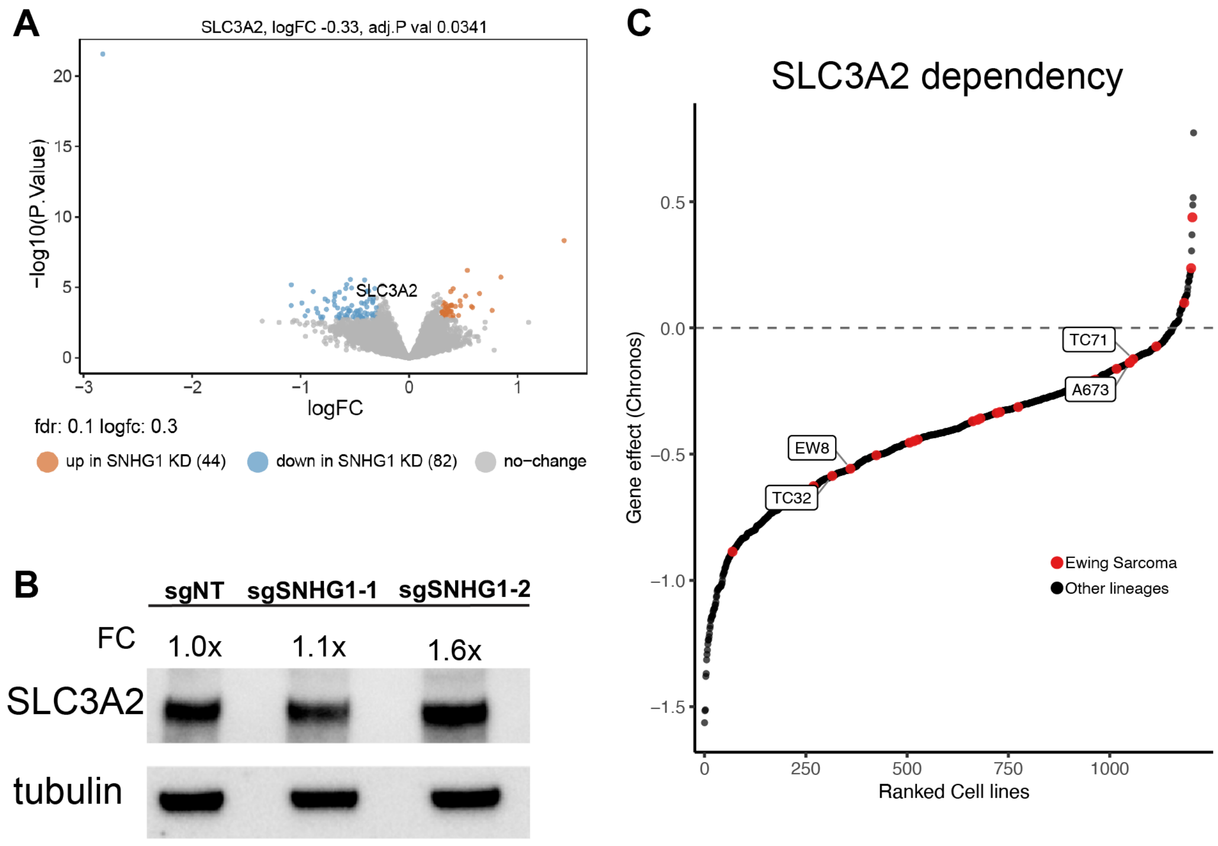

Supplementary Figure S14. SLC3A2. (**A**) Volcano plots showing differential expressed genes in A673 cells with SNHG1 knockdown after dCas9-KRAB induction with Dox. (**B**) Western blot of SLC3A2 protein in A673 dCas9-KRAB cells transduced with indicated sgRNAs. Tubulin was used as a loading control. (**C**) Gene effect scores for SLC3A2 across the 1208 cell lines in CRISPR (Chronos) DepMap 26Q1 data.

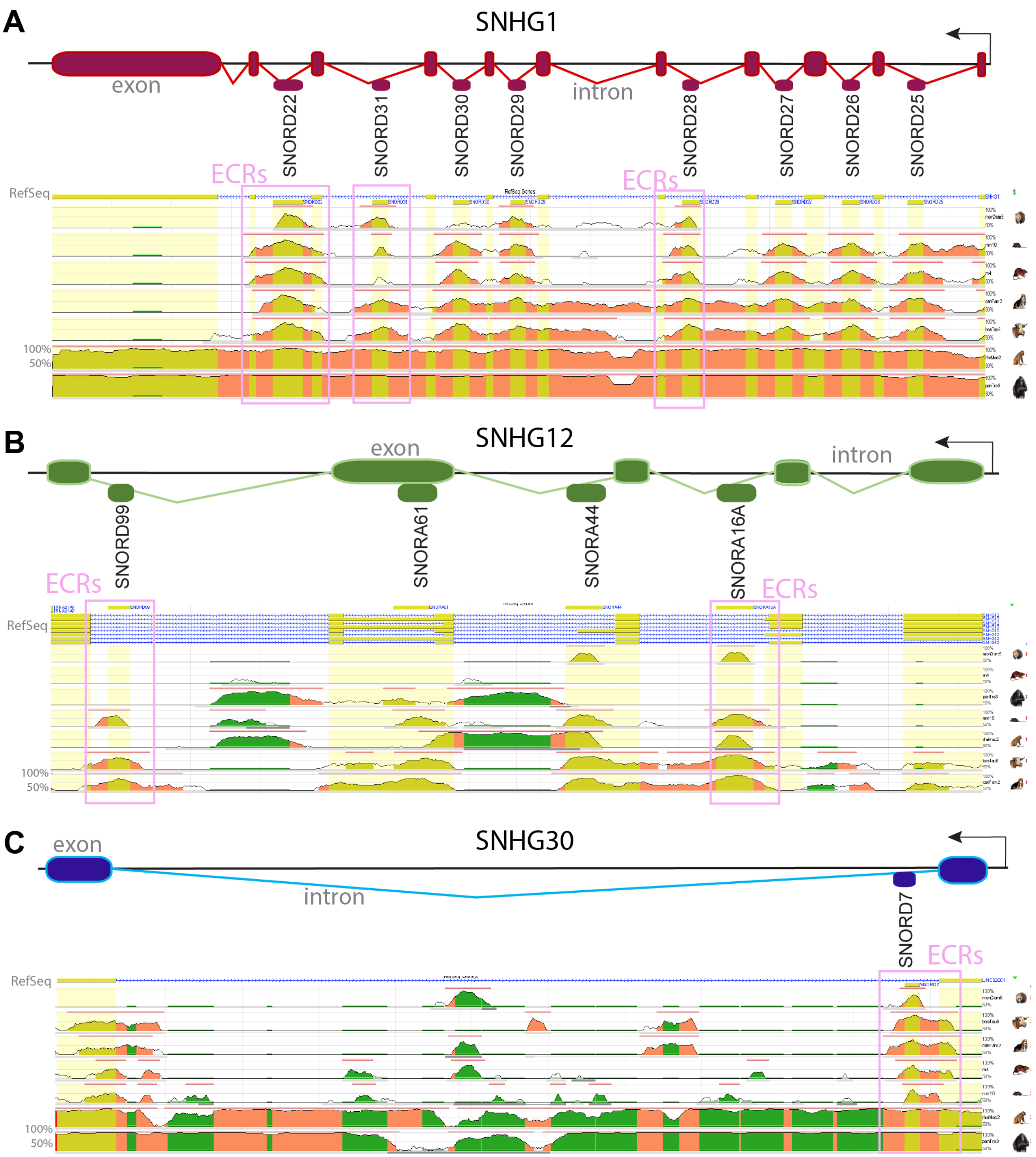

Supplementary Figure S15. Conserved regions in SNHG1, SNHG12 and SNHG30 genes. The sequence similarity for (**A**) SNHG1, (**B**) SNHG12 and (**C**) SNHG30 between humans and other vertebrates was analyzed by the ECR Browser. The Evolutionary Conserved Regions (ECRs) across seven species are indicated by pink lines and highlighted by pink rectangles.

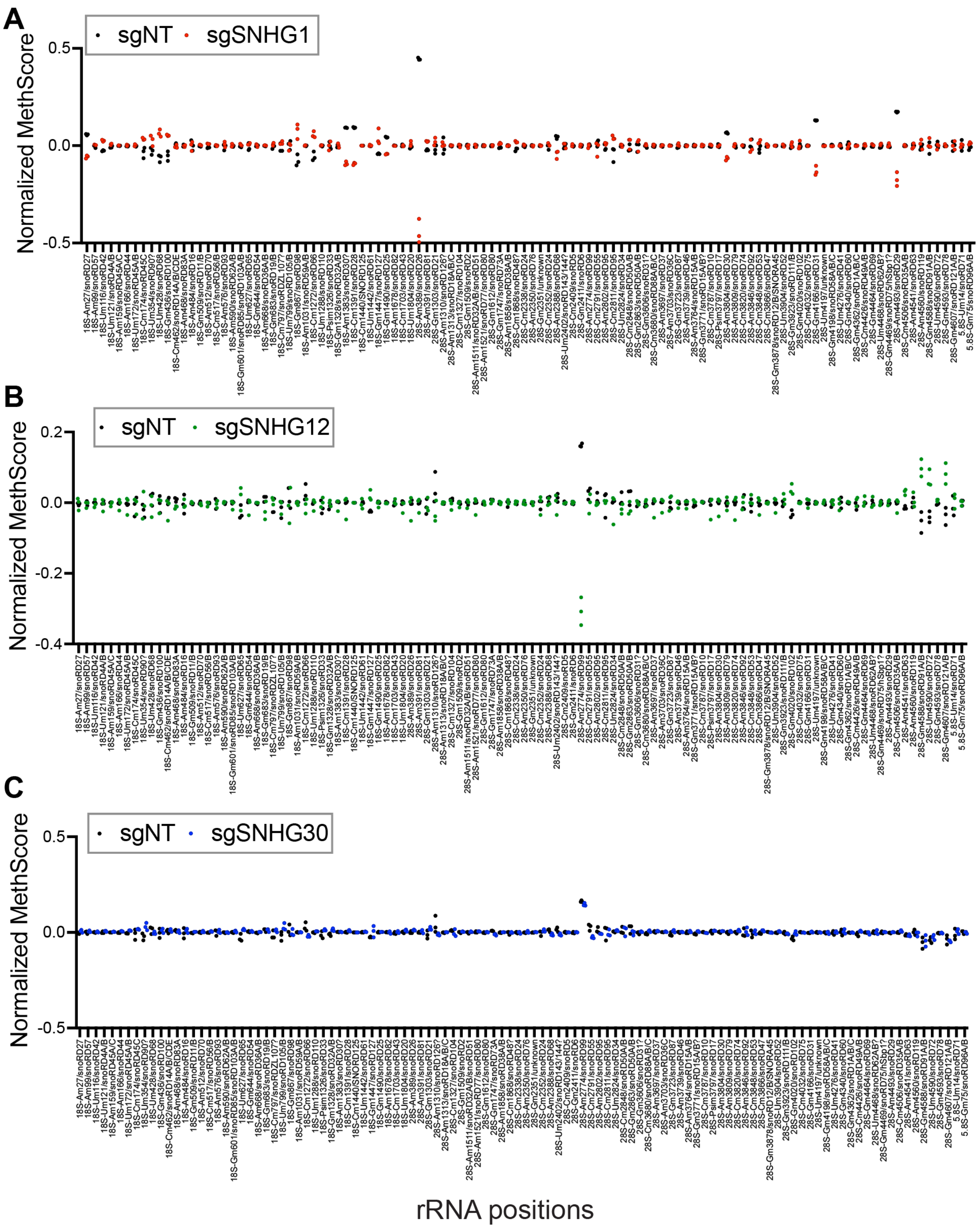

Supplementary Figure S16. RiboMethSeq Profile of Ewing SNHGs KD cells. The 2'-O-Methylation levels at each known modified nucleotide site in 18S, 28S, and 5.8S rRNA evaluated by RiboMethSeq. Normalized Meth Scores are plotted for A673 comparing sgNT controls with sgSNHG samples. (**A**) sgSNHG1 KD cells relative to sgNT cells. (**B**) sgSNHG12 KD cells relative to sgNT cells. (**C**) sgSNHG30. (n = 3 biological replicates).

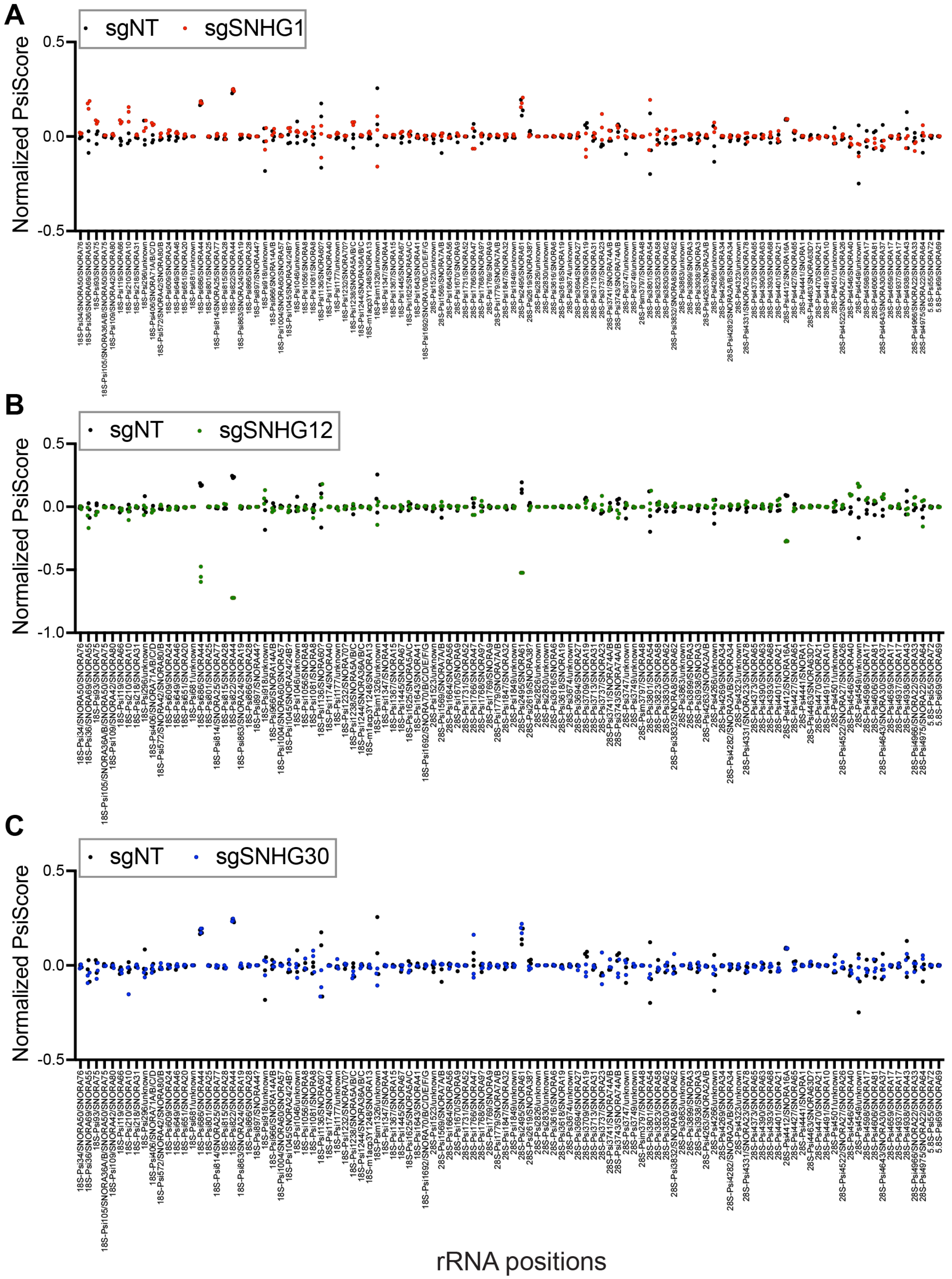

Supplementary Figure S17. HydraPsiSeq Profile of Ewing SNHGs KD cells. The pseudouridylation levels at each known modified nucleotide site in 18S, 28S, and 5.8S rRNA evaluated by HydraPsiSeq. Normalized Psi Scores are plotted for A673 comparing sgNT controls with sgSNHG samples. (**A**) sgSNHG1 KD cells relative to sgNT cells. (**B**) sgSNHG12 KD cells relative to sgNT cells. (**C**) sgSNHG30. (n = 3 biological replicates).

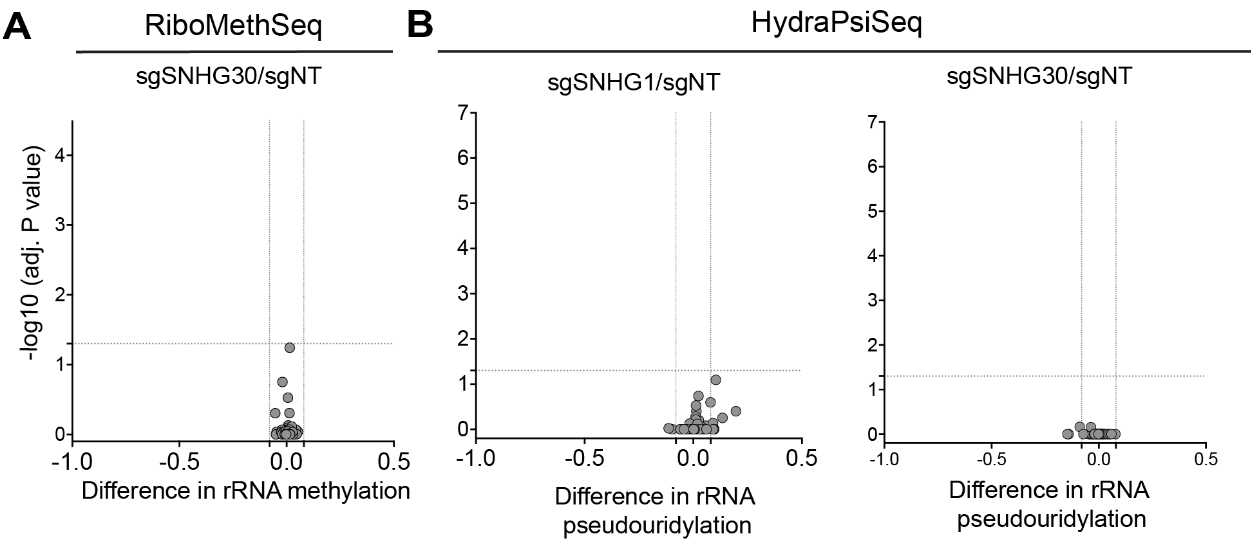

Supplementary Figure S18. Significant post-transcriptional modifications in rRNAs in SNHGs KD cells. (**A**) rRNA 2'-O-methylation by RiboMethSeq comparing sgNT controls with sgSNHG30 (**B**) Psi Scores values for the rRNA positions with significant difference of Psi Score between SNHG1 KD or SNHG30 KD and controls. Statistically significant differences in rRNA methylation or pseudouridylation are defined as absolute of difference > 0.08 and adj. p-value < 0.05. Adjusted p-values are from multiple unpaired t tests, followed by Holm-Sidak's multiple comparisons test. n = 3.

A

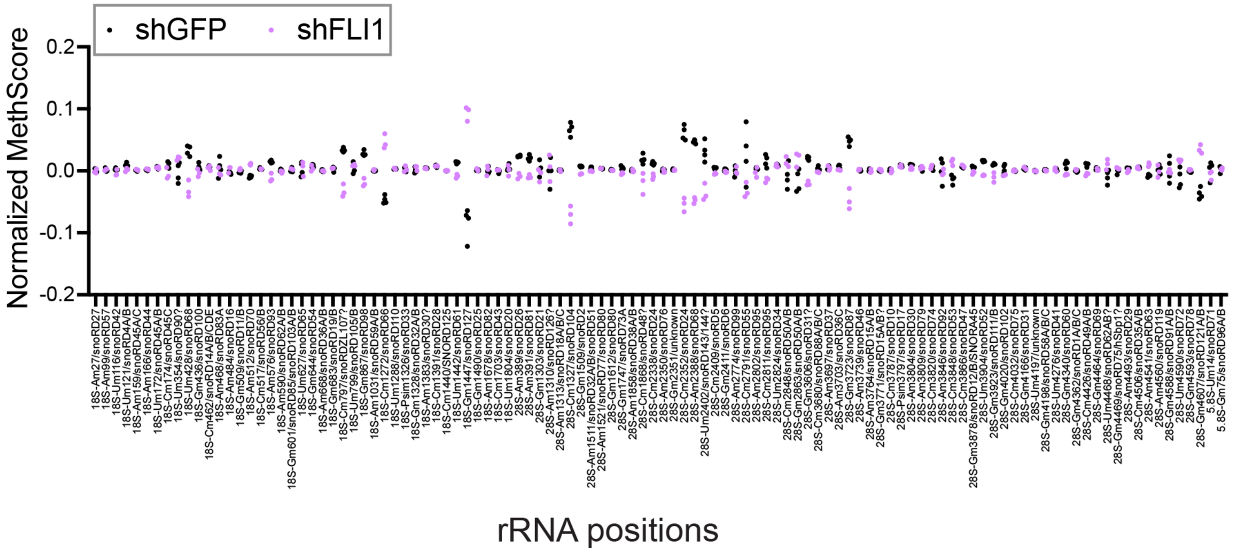

Supplementary Figure S19. RiboMethSeq Profile of Ewing shFLI1 KD cells. The 2’-O-Methylation levels at each known modified nucleotide site in 18S, 28S, and 5.8S rRNA were evaluated by RiboMethSeq. Normalized Meth Scores are plotted for A673 comparing shGFP controls with shFLI1 samples. (**A**) shFLI1 KD cells relative to shGFP cells.
